# Mortality patterns related to stroke and dementia among older adults in the U. S.: a CDC WONDER analysis from 1999 to 2024

**DOI:** 10.3389/fneur.2026.1889772

**Published:** 2026-08-26

**Authors:** Maryam Saghir, Eshal Saghir, Maliha Khalid, Muhammad Umar, Sahil Jairamani, Areej Dar, Hasiba Karimi, Muhammad Khalid Afridi, Syed Ijlal Ahmed, Jamal S. Rana, Elangovan Krishnan, Ishtiaq Ahmad, Suleman Sofian Suliman

**Affiliations:** 1 Department of Medicine, Jinnah Sindh Medical University, Karachi, Pakistan; 2 Department of Medicine, Dow University of Health Sciences, Karachi, Pakistan; 3 Khairpur Medical College, Khairpur, Pakistan; 4 Liaquat University of Medical and Health Sciences, Jamshoro, Pakistan; 5 Shaikh Khalifa Bin Zayed Al-Nahyan Medical & Dental College, Rahim Yar Khan, Lahore, Pakistan; 6 Faculty of Medicine, Bezmialem Vakif University, İstanbul, Türkiye; 7 Saint Francis Medical Center, Monroe, MO, United States; 8 Department of Cardiology, Kaiser Permanente Northern California, Oakland, CA, United States; 9 Health Sciences Center, University of Louisville School of Medicine, Louisville, KY, United States; 10 University of Iowa Health Care, Iowa City, IA, United States; 11 Faculty of Medicine and Surgery, National University-Sudan, Khartoum, Sudan

**Keywords:** dementia, epidemiology, mortality trends, retrospective analysis, stroke

## Abstract

**Introduction:**

Blood flow impairment due to stroke substantially causes neuronal loss, ultimately leading to cognitive decline. Dementia is a progressive neurocognitive disorder that represents a major public health challenge worldwide.

**Methods:**

We analyzed stroke and dementia related mortality trends among older adults using the Centers for Disease Control and Prevention Wide-Ranging Online Data for Epidemiologic Research (CDC WONDER) database from 1999 to 2024. Age-Adjusted Mortality Rates (AAMRs) per 100,000 were calculated and categorized by demographics and region. Joinpoint regression was used to estimate Annual Percent Change (APC) and Average Annual Percent Change (AAPC).

**Results:**

A total of 965,793 deaths due to stroke and dementia among older adults aged ≥65 years were recorded. Among the subgroups of dementia, unspecified dementia reported the greatest mortality. The AAMR observed a steep incline from 69.34 in 1999 to 99.25 in 2024. Older females observed higher overall AAMR than older males. Racially, Non-Hispanic Black population had the highest overall AAMR. Regionally, the highest overall AAMR was recorded by the Southern U. S. Despite COVID-19, the post pandemic era observed increasing mortality.

**Conclusion:**

Our cohort exhibited a rise in mortality rates among elderly with stroke and dementia, underscoring the need for preventive strategies, and targeted interventions in vulnerable populations and regions.

## 1 Introduction

Stroke, an acute neurological condition caused by a sudden interruption of cerebral blood flow leading to brain tissue injury. Meanwhile dementia is a chronic and progressive condition of the neurocognitive system characterized by a decline in memory and executive functioning. Both of these conditions are two of the most major causes of mortality among the older adults in the United States (U. S.) for many years and carry the burden of mortality and morbidity (1, 2). Their combined burden has grown increasingly complex over the years as the demographic landscape has shifted the past quarter century (3). As the life expectancy is rising and the baby boomer generation is aging into late adulthood, the number of Americans aged 65 years and older has expanded rapidly generating higher population level exposure to neurological diseases. Long-term surveillance of stroke and dementia mortality has become essential for assessing progress, identifying disparities and understanding how the health system changes over time (4).

Previous epidemiological studies have described National trends in stroke mortality and age adjusted mortality rates in the United States (5, 6). However few studies have comprehensively evaluated long term mortality involving stroke together with specific dementia subtypes including Alzheimer’s disease (AD), vascular dementia (VaD), and unspecified dementia, using a multiple cause of death (MCD) approach while simultaneously examining demographic and geographic disparities over a 25 year period (2).

Stroke has been ranked among the leading causes of death, especially within the elderly population (7). After steep declines in stroke mortality from late 1990s through the early 2010s, driven by improved hypertension control, better acute stroke care, reduction in smoking, and expanded use of lipid lowering and antithrombotic therapies, all indicate that these improvements have plateaued (8). The downward trend has slowly but dramatically increased in stroke related modalities in certain populations that have been reported (9). Persistent disparities in cardiovascular risk factors and rising prevalence of obesity and diabetes, along with uneven access to preventative care have contributed to these shifts. As a result, once characterized by consistent improvement, the trajectory of stroke mortality now reflects a more nuanced and uneven landscape. At the same time, advances in acute stroke management, including thrombolysis, mechanical thrombectomy and improved secondary prevention, have increased stroke survival, resulting in a growing population of survivors at increased risk of developing subsequent cognitive impairment and dementia.

Mortality involving AD, VaD, and unspecified dementia has exhibited a long term upward trajectory across the same period. In addition, increasing Diagnostic awareness and improvements in cognitive assessments, evolving International Statistical Classification of Diseases and Related Health Problems-10th Revision (ICD-10) coding practices and changes in death certification may have influenced reported dementia related mortality over time. Its risk increased sharply with advancing age, and the rapid growth of the oldest population aged more than 85 has amplified mortality rates (10). Unlike stroke, where advances in acute treatment and secondary prevention strategies have reduced case fatality, dementia still lacks disease modified therapies that substantially alter disease progression or mortality (11, 12). Dementia mortality, meanwhile, increased sharply during the COVID-19 pandemic. Rising by more than 10% between 2019 and 2020 before declining slightly but still remaining above the pre-pandemic levels (13). For stroke, COVID-19 influenced mortality through multiple pathways: delays in emergency presentation; reduced access to time-sensitive interventions such as thrombolysis and thrombectomy (14). For these reasons, multiple cause of death (MCD) analysis, which considers both the underlying cause of death and all the contributing conditions recorded on the death certificate, offers more comprehensive representation of mortality patterns in late life than analysis based solely on the underlying cause of death.

The Centers for Disease Control and Prevention Wide-Ranging Online Data for Epidemiologic Research (CDC WONDER) provides nationally representative mortality data spanning more than two decades, enabling comprehensive evaluation of temporal trends across demographic groups, and causes of death (15). The 1999 till 2024 period captures major transitions in clinical practice of mechanical thrombectomy, improved acute stroke pathways, rapid growth in electronic health record use, and expanded dementia awareness alongside societal and epidemiological factors that had an especially profound impact on stroke, and dementia mortality. Using CDC WONDER MCD, this study evaluates long term mortality trends involving stroke together with AD, VaD, and unspecified dementia from 1999 to 2024. In addition, it examines demographic and geographic disparities to provide a comprehensive assessment of mortality patterns and generates evidence to inform future healthcare planning policy and resource allocation.

## 2 Methods

### 2.1 Study design and data source

We conducted a retrospective, population based analysis using death certificate data retrieved from the CDC WONDER MCD record section of the database and analyzed data for older adults aged 65 and older, spanning January 1, 1999 to December 31, 2024 to assess long term mortality trends in stroke and dementia, using the (ICD-10) as follows: I60-I69 for stroke and F01, F03, and G30 for dementia, co-listed on the death certificates, within the MCD fields. The underlying cause of death (UCD) refers to the disease or condition that initiated the sequence of events leading to death, while MCD includes all other diseases or conditions that contributed to it (16). This methodology was employed as it captures a broader spectrum of the disease burden. The definitions of stroke and dementia used in this study are consistent with prior U. S. epidemiologic publications examining these conditions (17, 18). These dementia codes were selected because they represent major dementia-related ICD-10 categories available in CDC WONDER and allow consistency with prior mortality studies using death certificate data. We followed the guidelines established by the Strengthening the Reporting of Observational Studies in Epidemiology (STROBE) reporting standards (19). A completed STROBE checklist is provided in online Appendix 1. Our study did not require institutional review board approval as it used de-identified public-use data provided by the government.

Deaths were eligible for inclusion if the decedent was aged 65 years or older, the death occurred between January 1, 1999, and December 31, 2024, and the death certificate contained ICD-10 codes for stroke (I60–I69) together with one of the selected dementia categories (F01, F03, or G30) within the Multiple Cause of Death fields. Deaths occurring outside the study period, among individuals younger than 65 years, or not meeting the prespecified ICD-10 case definition were not included. The primary analyses included annual mortality counts, crude mortality rates, age-adjusted mortality rates, demographic and geographic subgroup analyses, place-of-death analyses, and Joinpoint trend analyses. Each study year represented a complete calendar year from January 1 through December 31.

Given the substantial healthcare disruptions during the COVID-19 pandemic, a sensitivity analysis was performed, to further investigate mortality trends beyond 2020 and assess potential pandemic-related impacts on mortality patterns. Trends in stroke and dementia-related mortality were analyzed by stratifying the study period across three periods: pre-COVID-19 (1999–2019), during COVID-19 (2020–2021), and post-COVID-19 (2022–2024).

### 2.2 Data abstraction

Datasets were retrieved and stratified according to population sizes, demographics (age, sex, race/ethnicity), and geography (urban–rural status, states, region, and place of death) from the CDC WONDER database. Age was categorized into three groups (65–74 years, 75–84 years, and ≥85 years) available within the CDC WONDER database to maintain consistency across analyses and facilitate comparisons between age groups (20). Sex included males and females. Racial and ethnic classifications followed CDC WONDER standard categories, and were classified into two groups, Hispanic or Latinos and the Non-Hispanics (NH) that included NH White, NH Black or African American, NH American Indian or Alaskan Native, and NH Asian or Pacific Islander. Urban–rural status was assigned using the National Center for Health Statistics Urban–Rural Classification Scheme. Urban areas had a population of >50,000, while rural areas had a population of <50,000, per the 2013 U. S. census classification (21). However, due to historical constraints in WONDER stratifications, urban–rural data were consistently available and examined only during the years 1999 to 2020. Geographic regions were stratified into Northeast, Midwest, South, and West, following U. S. Census Bureau guidelines (22). Medical facilities, hospice, home, and nursing home/long-term care facilities were the reported locations of deaths. Mortality trends were assessed by dementia categories to examine differences in mortality rates across dementia subtypes among stroke patients.

### 2.3 Statistical analysis

Annual crude mortality rates and age-adjusted mortality rates (CMRs and AAMRs) per 100,000 population, along with 95% confidence intervals (CIs), were obtained directly from the CDC WONDER database for each calendar year from 1999 to 2024 (1999–2020 for urban–rural analyses) to analyze changes in mortality patterns of stroke and dementia (23). CMRs were based on the number of deaths meeting the specified query criteria divided by the corresponding population for each calendar year, while AAMRs were calculated by CDC WONDER using the direct method of age standardization to the 2000 U. S. standard population.

Joinpoint Regression Program (Version 5.4.0.0, National Cancer Institute) was used to determine temporal trends in CMRs and AAMRs by estimating the annual percent change (APC) with corresponding 95% CIs by fitting log-linear regression segments at connected joinpoints. This software uses Monte Carlo permutation tests to determine the optimal number of join points (up to four), ensuring the best model fit while balancing model flexibility with parsimony, thereby allowing identification of significant changes in AAMR over time. APCs were considered increasing or decreasing if the slope describing the change in mortality was significantly different from zero, as determined by two-sided t-tests. Average annual percentage changes (AAPC) with 95% CIs were computed to summarize overall mortality trends over the course of the study. A *p*-value of less than 0.05 indicated statistical significance (24). To provide a more thorough understanding of the mortality burden, a comparative analysis was performed to assess whether the trend in AAMRs for stroke-related mortality differed significantly from the trend in AAMRs for stroke- and dementia-related mortality, and whether the trend in AAMRs for dementia-related mortality differed significantly from the trend in AAMRs for stroke- and dementia-related mortality.

## 3 Results

**Figure 1:**
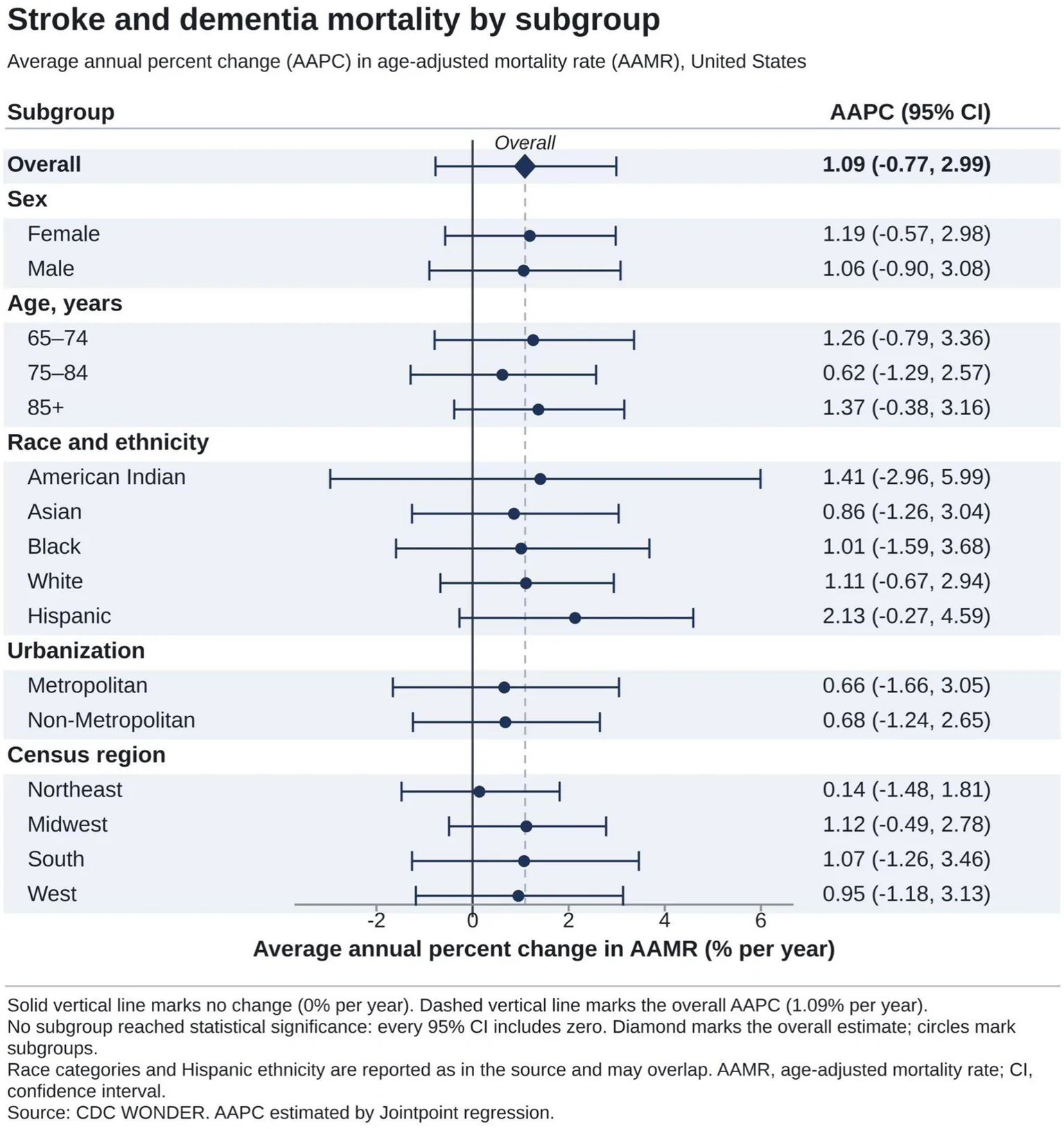
Average annual percent change (AAPC) of stroke and dementia–related age-adjusted mortality rates per 100,000 in older adults in the United States, 1999 to 2024.

From 1999 to 2024, deaths related to stroke and dementia accounted for 965,793 deaths among older adults (aged >65 years) in the U. S. Most deaths were recorded from nursing homes/long-term care settings (51%), with lower frequencies in the decedent’s home (20.25%), medical facilities (16.78%), and hospice settings (5.4%; Figure 1; Supplementary Tables 1, 2).

### 3.1 Annual trends

**Figure 2:**
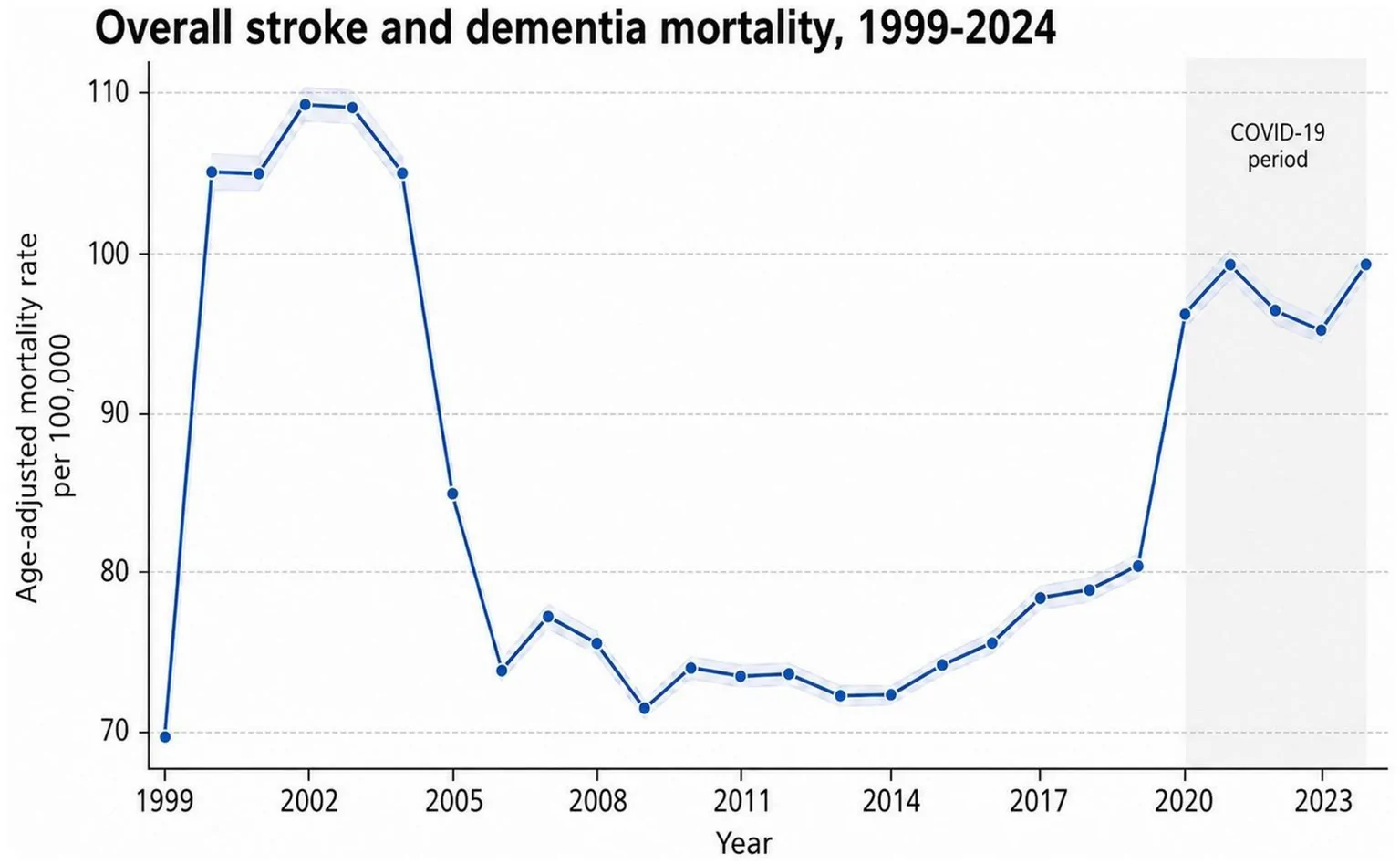
Annual trends stroke and dementia**-**related AAMRs per 100,000 in older adults aged ≥65 years in the United States 1999–2024.

The overall AAMR increased from 69.34 (95% CI: 68.45 to 70.22) in 1999 to 99.25 (95% CI: 98.39 to 100.11) in 2024. From 1999 to 2001, AAMR increased sharply (APC: 24.72; 95% CI: 0.27 to 55.13; *p* = 0.04), followed by a significant decline from 2001 to 2009 (APC: −6.75; 95% CI: −9.2 to −4.23; *p* < 0.01), before increasing again through 2024 (APC: 2.63; 95% CI: 1.82 to 3.44; *p* < 0.01; Supplementary Tables 3, 4; Figure 2).

### 3.2 Sex analysis

**Figure 3:**
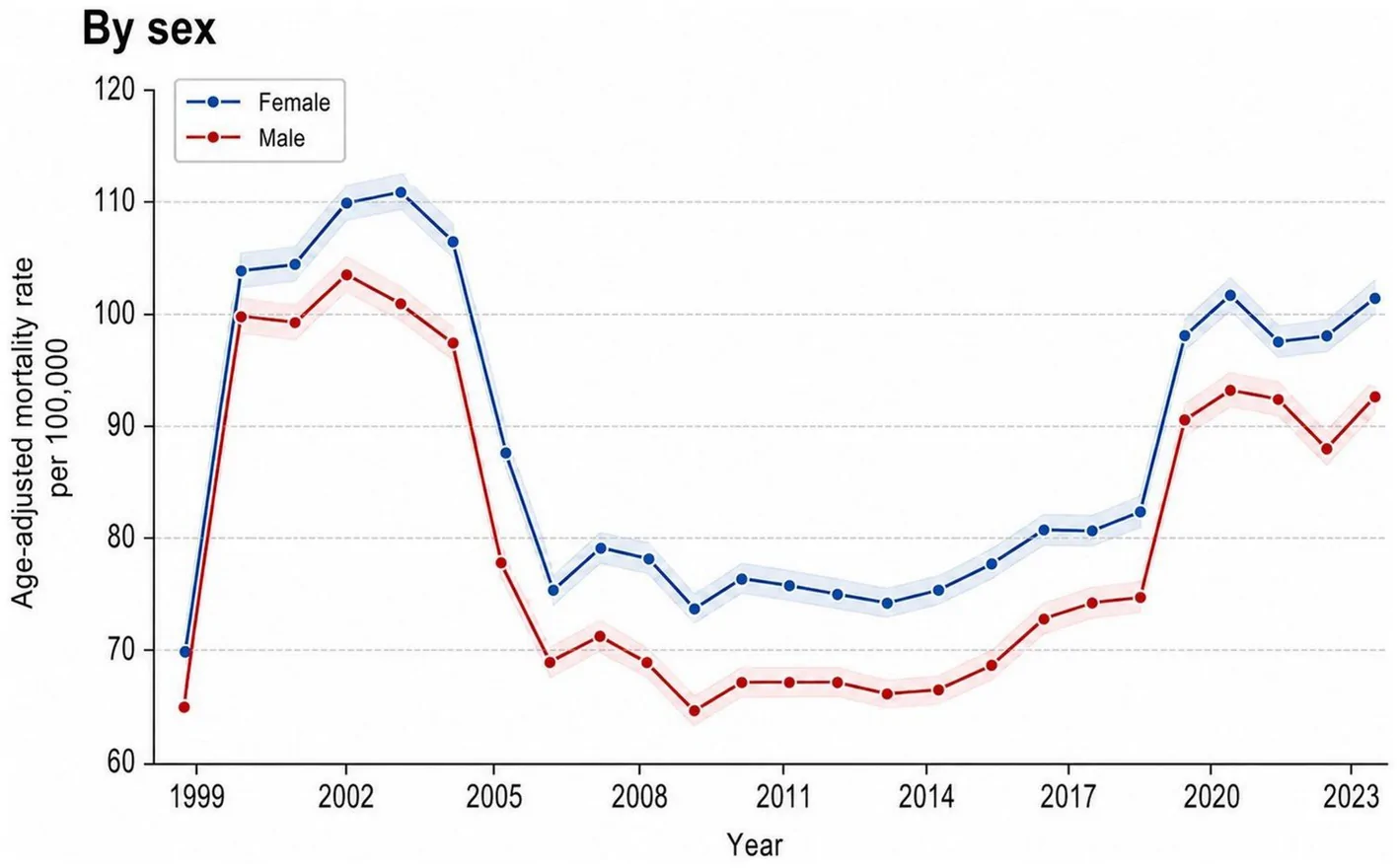
Stroke and dementia**-**related AAMRs per 100,000 stratified by sex in older adults aged ≥65 years in the United States 1999–2024.

Throughout the study period, AAMR was consistently higher among older females than older males, increasing from 70.28 (95% CI: 69.19 to 71.37) in 1999 to 102.4 (95% CI: 101.27 to 103.52) in 2024, while in older males, AAMR rose from 65.47 (95% CI: 63.98 to 66.95) to 93.11 (95% CI: 91.8 to 94.42) across the same period (Figure 3; Supplementary Tables 3, 4).

AAMR among older females increased significantly from 1999 to 2001 (APC: 23.1, 95% CI: 0.02 to 51.51; *p* = 0.04), followed by a decline from 2001 to 2010 (APC: −5.6, 95% CI: −7.56 to −3.59; *p* < 0.01), before increasing significantly through 2024 (APC: 2.89; 95% CI: 2.02 to 3.76; *p* < 0.01). Older male AAMR showed a period of non-significant increase from 1999 to 2001 (APC: 25.18; 95% CI: −0.8 to 57.98; *p* = 0.05), followed by a sharp decrease from 2001 to 2009 (APC: −7.24; 95% CI: −9.8 to −4.61; *p* < 0.01), and a significant increase from 2009 to 2024 (APC: 2.83, 95% CI: 2.01 to 3.64; *p* < 0.01).

### 3.3 Race analysis

**Figure 4:**
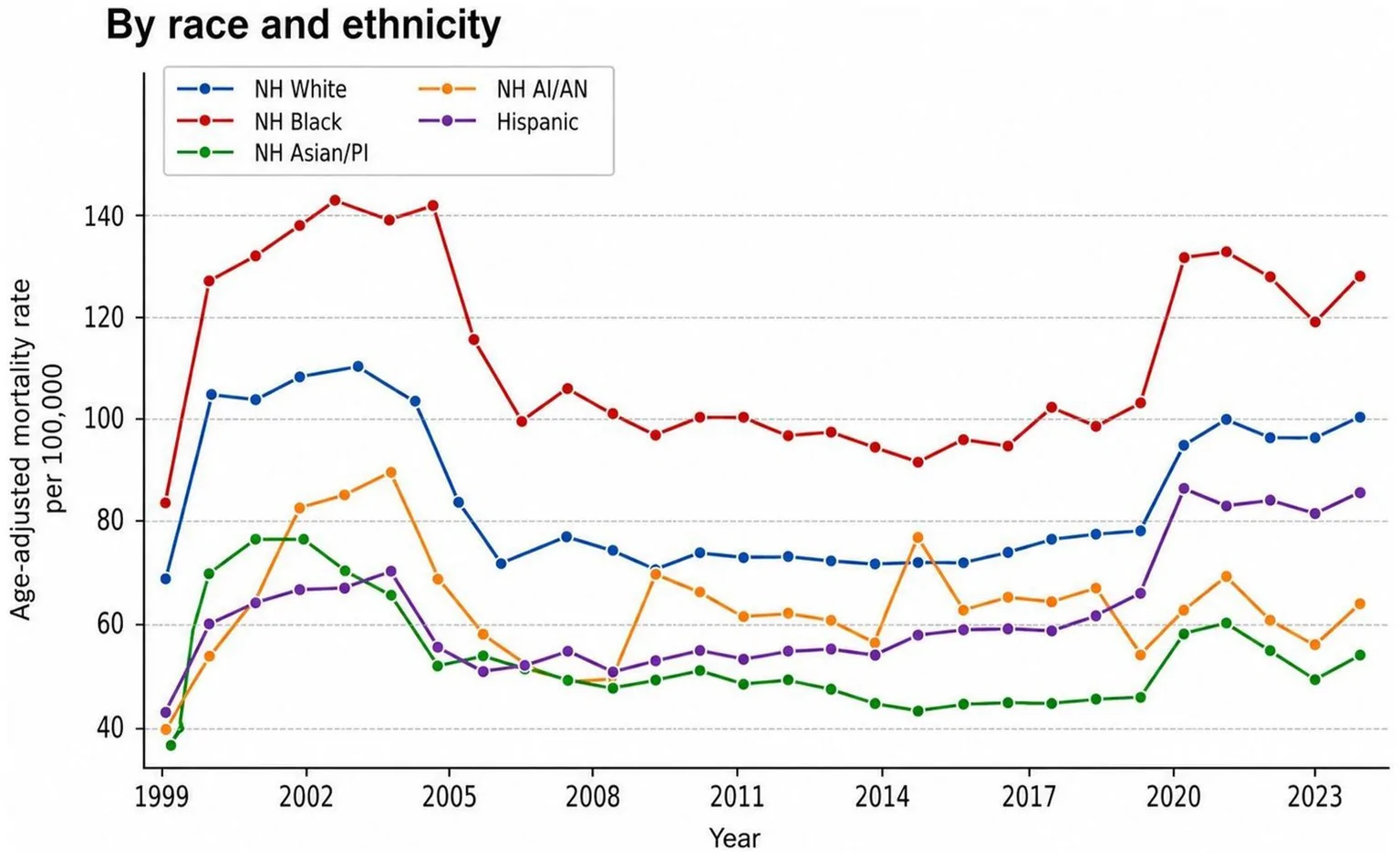
Stroke and dementia**-**related AAMRs per 100,000 stratified by race in older adults aged ≥65 years in the United States 1999–2024.

When stratified by race, NH Black individuals exhibited the highest AAMRs, increasing from 82.97 in 1999 to 127.98 in 2024, followed by NH White individuals (1999: 69.9 to 2024: 101.08), NH American Indian individuals (1999: 42.58 to 2024: 64.4), Hispanic individuals (1999: 42.3 to 2024: 86.1), and NH Asian individuals (1999: 36.54 to 2024: 56.29; Figure 4; Supplementary Tables 3, 5).

Among NH American Indian or Alaska Native population, AAMR increased significantly from 1999 to 2003 (APC: 19.99; 95% CI: 5.04 to 37.07; *p* < 0.01), followed by a non-significant decrease from 2003 to 2006 (APC: −12.1; 95% CI: −37.88 to 24.37; *p* = 0.44). There was no evidence of a significant increase or decrease in AAMR from 2006 to 2024 (APC: 0.05; 95% CI: −0.92 to 1.04; *p* = 0.9).

AAMR for the NH White population surged between 1999 and 2001 (APC: 24.11, 95% CI: 0.74 to 52.9; *p* = 0.04) followed by a significant decline from 2001 to 2009 (APC: −6.89, 95% CI: −9.26 to −4.46; *p* < 0.01). This was succeeded by a significant increase between 2009 and 2024 (APC: 2.82, 95% CI: 2.01 to 3.63; *p* < 0.01).

NH Black/African American individuals demonstrated a marked increase in AAMR from 1999 to 2002 (APC: 18.12; 95% CI: 6.35 to 31.2; *p* < 0.01), followed by a steeper decline between 2002 and 2006 (APC: −10.18; 95% CI: −18.2 to −1.37; *p* < 0.01), and a period of relative stability until 2018 (APC: −0.47; 95% CI: −1.72 to 0.79; *p* = 0.42). This was followed by a non-significant increase from 2018 to 2021 (APC: 11.46; 95% CI: −4.54 to 30.15; *p* = 0.15), and a non-significant decrease through 2024 (APC: −2.84; 95% CI: −9.7 to 4.54; *p* = 0.4).

Hispanic/Latino individuals exhibited an initial significant increase in AAMR from 1999 to 2003 (APC: 10.6, 95% CI: 3.85 to 17.8; *p* < 0.01), followed by a non-significant decline from 2003 to 2006 (APC: −10.57, 95% CI: −24.26 to 5.59; *p* = 0.16), and a moderate, yet significant increase from 2006 to 2017 (APC: 1.09, 95% CI: 0.005 to 2.2; *p* = 0.04). From 2017 to 2020, a non-significant increase occurred (APC: 10.88, 95% CI: −0.25 to 23.25; *p* = 0.05), followed by a period of relative stabilization through 2024 (APC: 0.72, 95% CI: −2.11 to 3.64; *p* = 0.59).

In the NH Asian/Pacific Islander population, AAMR demonstrated a pronounced escalation from 1999 to 2001 (APC: 38.87; 95% CI: 12.39 to 71.59; *p* < 0.01), followed by a decrease from 2001 to 2006 (APC: −8.35; 95% CI: −12.74 to −3.75; *p* < 0.01), and a gradual but significant decline from 2006 to 2018 (APC: −0.95; 95% CI: −1.82 to −0.08; *p* = 0.03). This was succeeded by a non-significant increase from 2018 to 2021 (APC: 8.76; 95% CI: −1.27 to 19.81; *p* = 0.08), and a significant decrease until 2024 (APC: −4.16; 95% CI: −8.82 to −0.21; *p* = 0.04).

### 3.4 Age groups

**Figure 5:**
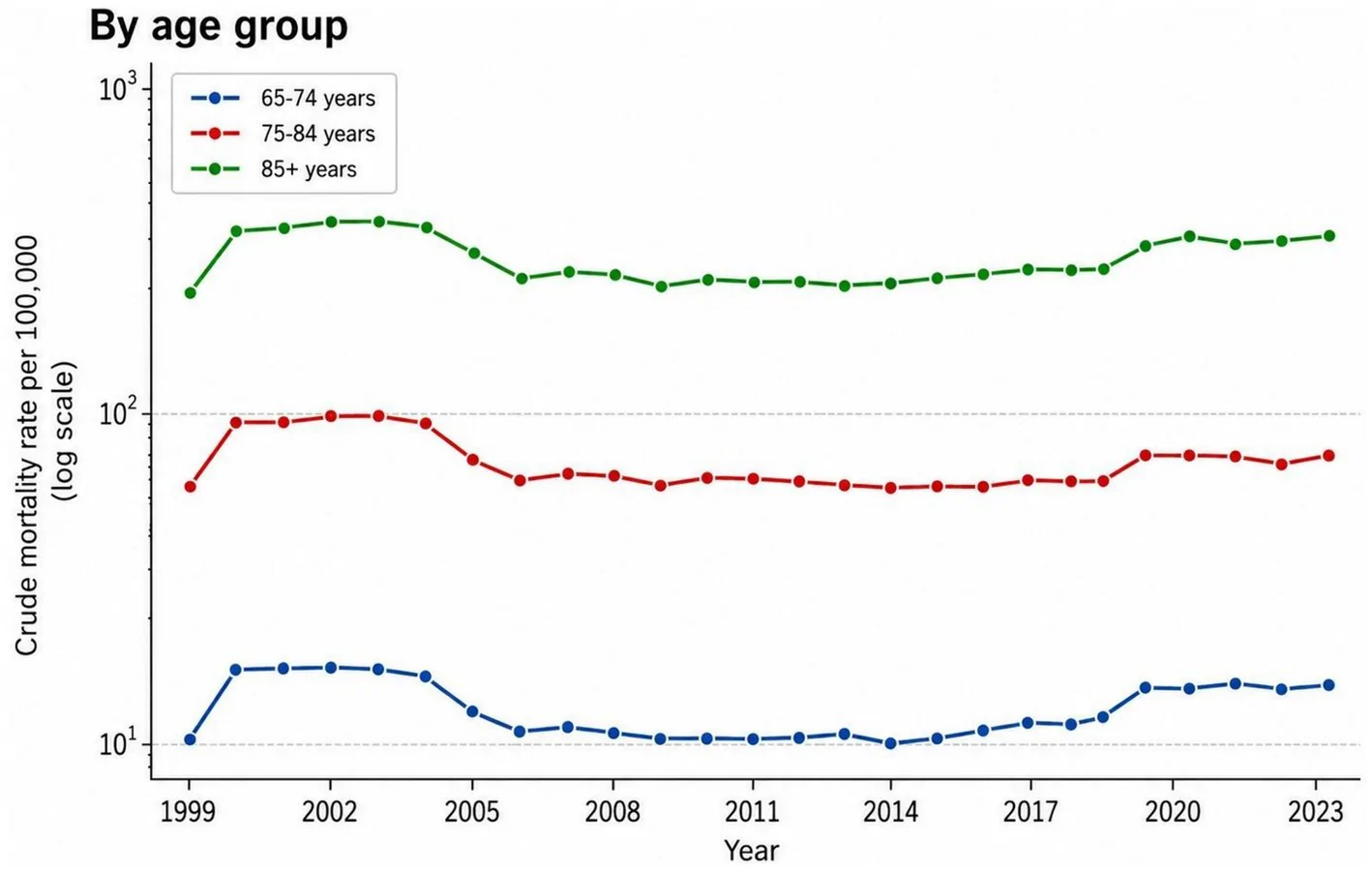
Stroke and dementia**-**related CMRs per 100,000 stratified by age in older adults aged ≥65 years in the United States 1999–2024.

Across the entire 1999–2024 interval, a consistent upward trend in CMR per 100,000 population was observed with increasing age. Older adults aged >85 years consistently exhibited the highest overall CMR, increasing from 322.8 in 1999 to 486.72 in 2024, followed by those aged 75–84 years (1999: 69.5 to 2024: 90.5), and the 65–74 year age group (1999: 9.72 to 2024: 14.2; Figure 5; Supplementary Tables 3, 6).

CMR among older adults aged 65–74 years, observed a non-significant increase from 1999 to 2001 (APC: 22.06, 95% CI: −4.16 to 55.46; *p* = 0.1), followed by a sharp decline from 2001 to 2010 (APC: −6.73, 95% CI: −9.08 to −4.33; *p* < 0.01). A modest rise was observed from 2010 to 2024 (APC: 3.95, 95% CI: 2.98 to 4.92; *p* < 0.01).

Among those aged 75–84 years, CMR increased non-significantly from 1999 to 2001 (APC: 22.95, 95% CI: −1.53 to 53.43; *p* = 0.06), followed by a decrease from 2001 to 2009 (APC: −6.52, 95% CI: −9.12 to −3.84; *p* < 0.01). This was followed by a subtle increase from 2009 to 2024 (APC: 1.89, 95% CI: 1.01 to 2.78; *p* < 0.01).

Among the 85 + year age group, CMR rose from 1999 to 2001 (APC: 24.14, 95% CI: 0.8 to 52.89; *p* = 0.04), followed by a drop between 2001 and 2010 (APC: −5.84, 95% CI: −7.74 to −3.9; *p* < 0.01). A significant increase followed from 2010 to 2024 (APC: 3.27, 95% CI: 2.45 to 4.09; *p* < 0.01).

### 3.5 Geographic region analysis

#### 3.5.1 Census region

**Figure 6:**
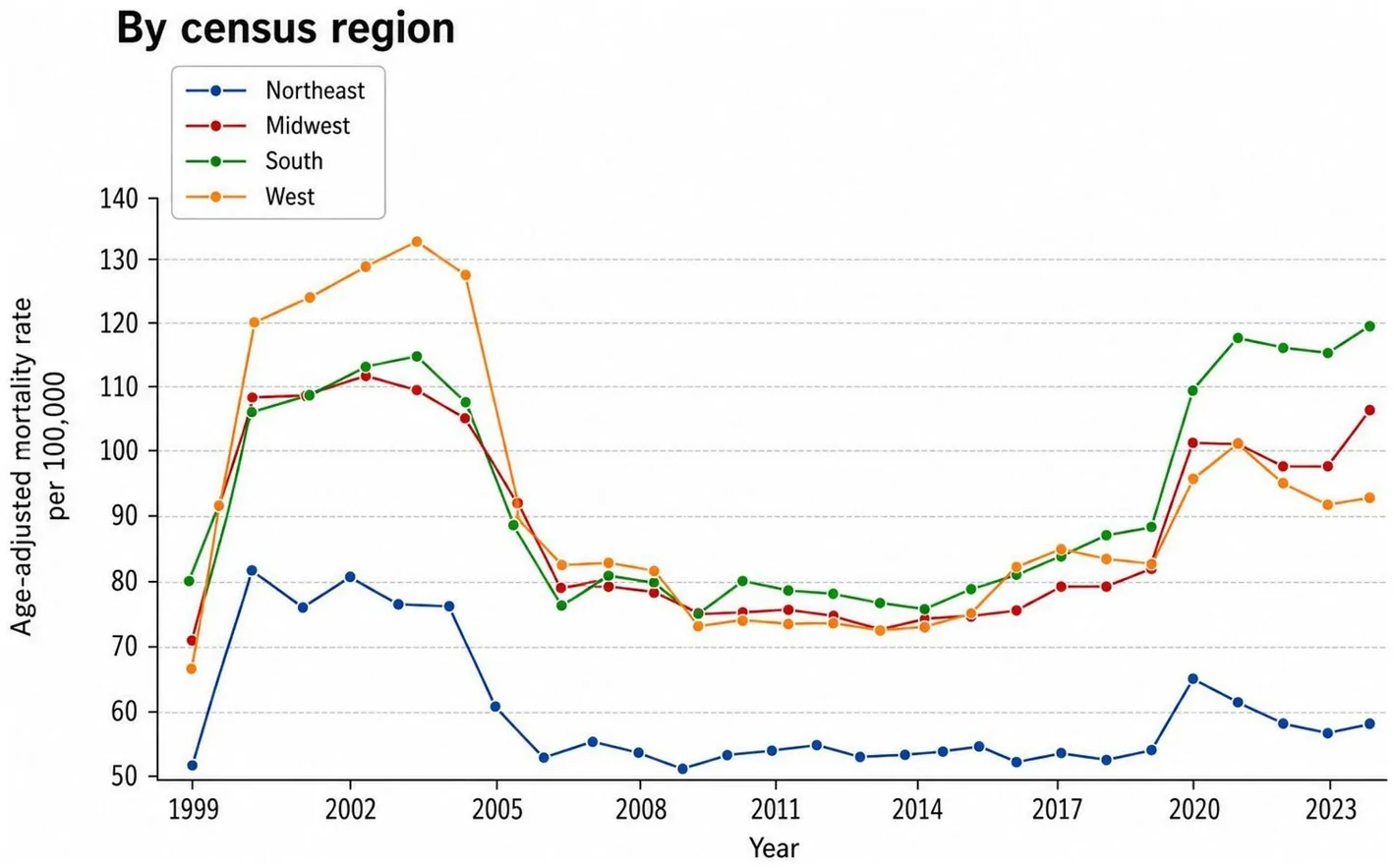
Stroke and dementia**-**related AAMRs per 100,000 stratified by census region in older adults aged ≥65 years in the United States 1999–2024.

Throughout the study period, older adults living in different U. S. Census Regions demonstrated persistent regional disparities in AAMRs for stroke and dementia-related mortality. The Southern region consistently bore the highest mortality burden with AAMR increasing from 79.74 in 1999 to 119.5 in 2024, followed by the Midwest (71.78 to 106.65), the West (67.33 to 92.52), and the Northeast (51.83 to 59.4; Figure 6; Supplementary Tables 3, 7).

In the Southern region, AAMR increased significantly from 1999 to 2001 (APC: 7.48; 95% CI: 1.8 to 13.47; *p* = 0.01), followed by a non-significant decrease from 2001 to 2006 (APC: −13.24; 95% CI: −26.5 to 2.39; *p* = 0.08), and a period of relative stabilization from 2006 to 2017 (APC: 0.18; 95% CI: −1.1 to 1.49; *p* = 0.75). This was followed by a surge from 2017 to 2021 (APC: 9.52; 95% CI: 2.3 to 17.26; *p* = 0.01), before stabilizing through 2024 (APC: 0.65; 95% CI: −5.3 to 6.98; *p* = 0.82).

The Midwest region demonstrated a sharp increase from 1999 to 2001 (APC: 21.85, 95% CI: 0.49 to 47.74; *p* = 0.04) followed by a significant decline from 2001 to 2011 (APC: −5.03, 95% CI: −6.58 to −3.47; *p* < 0.01), and a significant increase between 2011 and 2024 (APC: 3.14, 95% CI: 2.22 to 4.07; *p* < 0.01).

AAMR in the West surged from 1999 to 2001 (APC: 36.02, 95% CI: 5.52 to 75.32; *p* = 0.02) followed by a significant decline from 2001 to 2009 (APC: −8.17, 95% CI: −10.81 to −5.46; *p* < 0.01), and a moderate increase through 2024 (APC: 2.05, 95% CI: 1.15 to 2.95; *p* < 0.01).

In the Northeast, AAMR exhibited an initial significant increase from 1999 to 2001 (APC: 23.46, 95% CI: 3.01 to 47.97; *p* = 0.02) followed by a significant decrease from 2001 to 2007 (APC: −7.96, 95% CI: −11.43 to −4.34; *p* < 0.01). This was followed by a gradual increase from 2007 to 2024 (APC: 0.66, 95% CI: 0.06 to 1.27; *p* = 0.03).

#### 3.5.2 States

**Figure 7:**
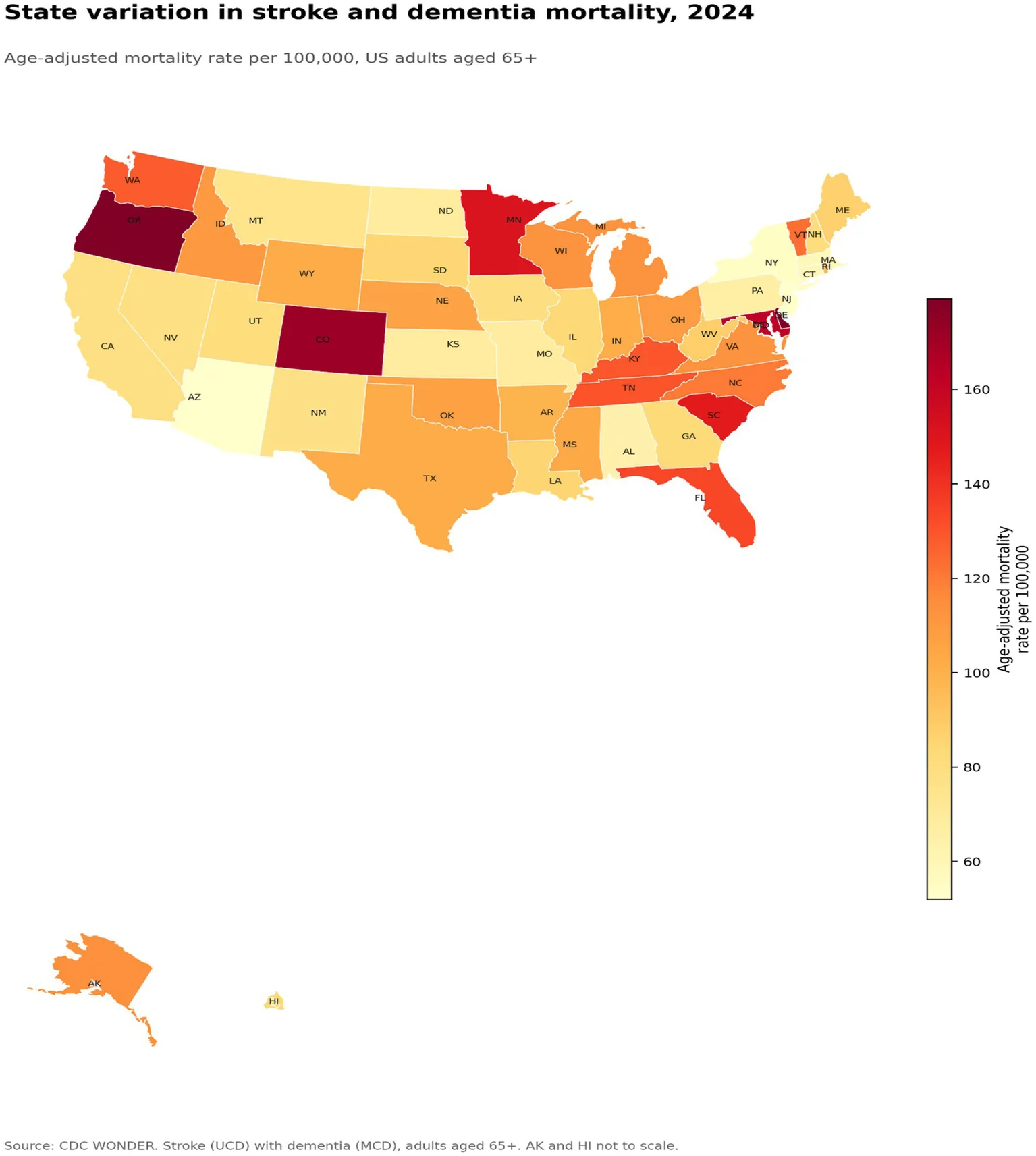
Stroke and dementia**-**related AAMRs per 100,000 stratified by states in older adults aged ≥65 years in the United States, 2024.

A notable disparity in AAMRs was observed among states, ranging from 47.38 (95% CI: 45.64 to 49.12) in New Jersey to 202.78 (95% CI: 197.38 to 208.18) in Oregon (Figure 7; Supplementary Table 8).

In 2024, states in the upper 90th percentile of AAMRs for stroke and dementia-related mortality included Minnesota, Maryland, Colorado, Delaware, and Oregon. These states had AAMRs that were more than three times higher than those in the lower 10th percentile, which included Connecticut, Massachusetts, New York, Arizona, and New Jersey.

#### 3.5.3 Urbanization

**Figure 8:**
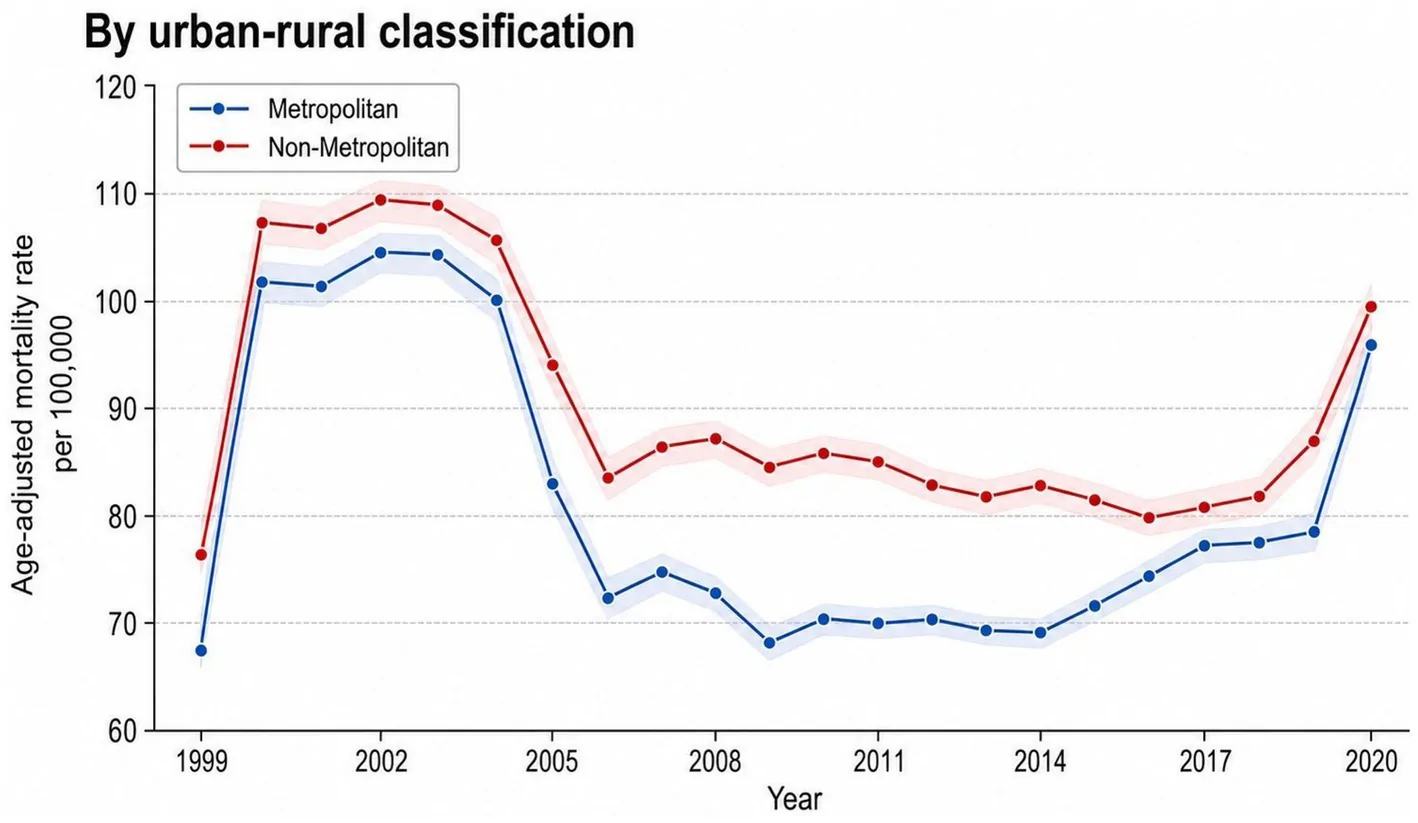
Stroke and dementia**-**related AAMRs per 100,000 stratified by urban–rural classification in older adults aged ≥65 years in the United States 1999–2020.

Rural areas consistently exhibited higher AAMRs than urban areas from 1999 to 2020. On average, rural areas had an AAMR of 90.88 (95% CI: 90.42 to 91.34) compared to urban areas reporting an AAMR of 80.56 (95% CI: 80.35 to 80.76; Figure 8; Supplementary Tables 3, 9).

Rural areas experienced a significant increase in AAMR between 1999 and 2002 (APC: 13.15; 95% CI: 5.02 to 21.91; *p* < 0.01), followed by a marked decline to 2006 (APC: −8.31; 95% CI: −14.36 to −1.82; *p* = 0.01). From 2006 to 2018, an extended period of relative stability was observed (APC: −0.7; 95% CI: −1.67 to 0.27; *p* = 0.14), followed by a non-significant increase through 2020 (APC: 10.73; 95% CI: −2.94 to 26.35; *p* = 0.11).

Urban areas demonstrated a non-significant increase from 1999 to 2001 (APC: 25.1; 95% CI: −0.91 to 57.95; *p* = 0.05), followed by a significant decline until 2009 (APC: −6.9; 95% CI: −9.5 to −4.23; *p* < 0.01). A moderate upward trend was observed from 2009 to 2020 (APC: 2.43; 95% CI: 1.05 to 3.83; *p* < 0.01).

#### 3.5.4 Annual trends for stroke-related AAMR

The overall stroke-related AAMR, declined from 1999 to 2002 (APC: −2.7; 95% CI: −5.33 to −0.01; *p* = 0.04), followed by a sharper decrease from 2002 to 2009 (APC: −5.73; 95% CI: −6.67 to −4.77, *p* < 0.01), and a gradual decrease through 2018 (APC: −0.81; 95% CI: −1.47 to −0.14; *p* = 0.02). Rates increased from 2018 to 2021 (APC: 5.83; 95% CI: 0.02 to 11.97; *p* = 0.04), before decreasing non-significantly through 2024 (APC: −1.72; 95% CI: −4.34 to 0.97; *p* = 0.18). Across the entire 1999–2024 interval, the overall mortality trend for stroke alone, maintained a decreasing trend (AAPC: −1.78, 95% CI: −2.57 to −0.99; *p* < 0.01), indicating that the long-term trend for stroke-related AAMR was significantly different from the combined stroke and dementia-related AAMR.

#### 3.5.5 Annual trends for dementia-related AAMR

The overall AAMR for dementia alone, demonstrated a non-significant increase from 1999 to 2001 (APC: 19.28; 95% CI: −0.14 to 42.48; *p* = 0.05), followed by a modest increase from 2001 to 2021 (APC: 0.96; 95% CI: 0.56 to 1.36; *p* < 0.01). A non-significant decline occurred from 2021 to 2024 (APC: −4.13; 95% CI: −9.69 to 1.77; *p* = 0.15). Over the entire 1999 to 2024 period, dementia-related mortality exhibited an overall increasing trajectory (AAPC: 1.68, 95% CI: 0.15 to 3.23; *p* = 0.03), suggesting a long-term trend distinct from that of combined stroke and dementia-related AAMR.

### 3.6 Dementia sub-types

Across the study period, the highest proportion of deaths across various types of dementia were found with unspecified dementia (57.57%), followed by VaD (21.99%), and AD (20.43%). Accordingly, the highest AAMRs were observed in unspecified dementia (53.21), while the lowest were observed in AD (18.75) and VaD (18.64) categories.

The various types of dementia among older adults with stroke, exhibited variable trends. VaD was the only subtype that exhibited the most pronounced increase (AAPC: 15.68, 95% CI: 11.2 to 20.34; *p* < 0.01). In comparison, AD-related deaths experienced a decrease (AAPC: −1.28; 95% CI: −2.05 to −0.5; *p* < 0.01), while unspecified dementia-related deaths exhibited a non-significant decline (AAPC: −1.32; 95% CI: −4.66 to 2.13; *p* = 0.44) throughout the study period (Supplementary Figure 1).

### 3.7 Pandemic era

The AAMRs for stroke and dementia during the peak COVID-19 years (2020–2021) were 20% higher than the pre-COVID period (1999–2019). Following the pandemic, death rates plateaued, with a minimal decrease of 0.88% in the post-COVID period (2022–2024). Overall, the burden remained substantially elevated post pandemic, with an overall increase of 18.94% compared to the pre-COVID period.

Stroke and dementia-related mortality demonstrated an overall increase, across all stratifications from the pre-pandemic to the pandemic period, followed by a slight reduction in the post-pandemic period. The most pronounced increases were observed among older adults aged 65–74 years and those aged 85 + years, with AAMRs increasing by 28.81 and 23.2% respectively, compared with a modest increase of 9.69% among those aged 75–84-year age group, from pre-pandemic to post-pandemic period. AAMRs for females were persistently higher than males increasing by 19.13 and 11.8%, respectively. The Hispanic population observed the steepest increase amongst all ethnicities, with rates escalating by 39.47%. Regionally, the South bore the highest burden, with a 33.84% rise in patients with stroke and dementia. All observed AAMR changes from the pre- to post-COVID period were statistically significant, indicated by non-overlapping 95% CIs (Supplementary Table 10).

## 4 Discussion

Our analysis reveals two diverging trends in stroke- and stroke-associated AD, VaD, and unspecified dementia-related mortality: stroke-related mortality has continued to decline, while mortality involving stroke and these dementia subtypes, particularly AD and VaD, has substantially increased among older adults. Because this study was based on multiple-cause-of-death data, these findings describe deaths in which both stroke and dementia were listed on the death certificate rather than establishing a causal relationship between the two conditions. Compared with previous epidemiological analyses describing temporal mortality trends, our study extends the current literature by simultaneously evaluating long-term national trends in deaths involving both stroke and specific dementia subtypes, examining demographic and geographic disparities. These findings provide additional insight into how population aging, improvements in stroke care, and evolving patterns of dementia-related mortality may collectively influence the future burden of cerebrovascular and neurodegenerative disease. The divergence of these trends, coupled with continued demographic and geographic disparities, underlines the need for broad public health approaches that address both vascular and neurodegenerative diseases. These findings describe deaths in which stroke and dementia were recorded on the death certificate rather than establishing a direct causal relationship between the two conditions. Furthermore, improvements in stroke prevention and acute management have enabled more individuals to survive their initial cerebrovascular event, thereby increasing the number of older adults living long enough to develop or die with coexisting neurodegenerative disorders. At the same time, continued population aging has substantially expanded the number of individuals at risk for both stroke and dementia, providing an important context for the observed divergence in mortality trends.

Sex-specific disparities are important to the understanding of mortality patterns. Generally, among those aged 65 and over, women have higher age-adjusted death rates for dementia in part due to women’s longer life expectancy (4, 25). In stroke mortality, women in the oldest age groups commonly have higher crude death rates that reflect both age and biological vulnerability (26, 27). Biologically, estrogen’s protective vascular role diminishes after menopause, potentially increasing their susceptibility to microvascular damage and subsequent cognitive decline (28). Social factors also play a role in that older women are more likely to live alone, have fewer socioeconomic resources, and serve as caregivers-all factors that may impede stroke recognition and reduce access to acute and long-term care (28). For their part, men tend to bear the burden of vascular risk factors and consequently a higher probability of adverse outcome-for example, hypertension, smoking, and coronary artery disease-that can enhance stroke threat earlier in adulthood (29). However, recent reduction in stroke fatality may reflect, in part, improvements in acute stroke care, prevention, and risk factor control, although these observational mortality data cannot establish the specific causes underlying these trends (30). In addition, increasing uptake of reperfusion therapies, specialized stroke unit care, improved secondary prevention, and better long-term management of hypertension, dyslipidemia, and atrial fibrillation have likely contributed to declining stroke mortality observed nationally, although these factors were not directly evaluated in the present analysis.

Racial and ethnic disparities in stroke and dementia mortality are a signal for systemic inequities. Stroke fatality is generally higher among older non-Hispanic Blacks, which may be related to the higher burden of hypertension, uncontrolled diabetes, and unequal access to quality care reported in previous studies (31). Common vascular risk factors may contribute to cognitive impairment and dementia disparities as reported previously (17). Geographic and urban–rural disparities further intensify these inequities, when considering that stroke mortality among older adults is higher in the U. S. South and in non-metropolitan areas; this can be ascribed to delays in access to care, fewer specialists, and a limited rehabilitation infrastructure (32). Historical mistrust, socioeconomic disadvantage, and underrepresentation of communities in clinical research have been proposed as important contributors to inequities in outcomes (33). Other communities with an increased risk include American Indian, Alaska Native, multiracial, and Hispanic communities, where structural and social determinants impede preventive care and the dementia regimen (34). Differences in educational attainment, health literacy, insurance coverage, access to specialist services, and the prevalence and control of vascular risk factors may further contribute to disparities in both stroke and dementia outcomes across racial and ethnic groups. These patterns have applicability in the world wherein ethnicity and social inequality significantly alter the cardiovascular and neurodegenerative disease burdens (7).

Age remains the most powerful threat factor for both stroke and dementia. Among U. S. older adults, stroke death rates sharply grow with age: for example, persons aged ≥ 85 years have much higher stroke mortality than those aged 65–74 (27). This is well documented in national vital statistics. Biological changes of aging-such as small vessel disease, amyloid accumulation, neuroinflammation, and decreasing neuroplasticity-contribute to heightened vulnerability with climbing age (35). Comorbid conditions such as atrial fibrillation, hypertension, diabetes, and frailty further increase vulnerability (36). Social realities, including isolation and limited access to care, further elevate mortality risk among the oldest-old, affecting stroke therapy and dementia management (37). The marked increase in mortality involving stroke and dementia among the oldest age groups should also be interpreted in the context of demographic changes within the U. S. population. Continued increases in life expectancy over recent decades have resulted in a larger proportion of adults surviving into advanced age, where both cerebrovascular disease and neurodegenerative disorders become substantially more prevalent. Consequently, population aging is likely an important contributor to the rising mortality burden observed in this study, independent of changes in disease incidence. These findings emphasize the importance of preventive strategies targeting vascular risk factors and healthy aging among older adults.

Geographic disparities in stroke- and dementia-related mortality reflect persistent healthcare inequalities. According to national data, the death rates for stroke among older adults in the U. S. South are the highest and those in the Northeast are the lowest. Factors contributing to these geographic differences include regional variation in vascular risk factor prevalence, for example hypertension, obesity, and diabetes, socio-economic status, and health care access. This issue is further compounded by rural–urban differences: older adults in rural counties often have delayed access to stroke centers, fewer neurologists, and limited rehabilitation infrastructure. A 2020 MMWR report noted that among persons who were aged 65 years or above, the nonmetropolitan stroke mortality rate was higher compared with the metropolitan areas (32). State-level analyses confirm disparities: states with historically high stroke mortality rates, such as many in the South-show slower deterioration, whereas states with strong preventive infrastructure display better outcomes (31). Regional differences in access to memory clinics, neurological services, rehabilitation programs, and primary care may similarly influence the recognition, management, and documentation of AD, VaD, and unspecified dementia on death certificates. These geographic disparities also call for policy interventions that should be tailored geographically, including telemedicine, mobile stroke units, and regional stroke networks.

Nationally, stroke mortality has fallen significantly over decades due to gains in prevention, acute care, and secondary prevention (38). A recent CDC WONDER analysis identified dramatic reductions in age-adjusted stroke mortality rates since the late twentieth century, although the pace of decline has slowed, and suggestive upward trends have manifested in recent years (29). Previous studies suggest that atrial fibrillation-associated strokes may contribute to these observed mortality patterns; however, this relationship cannot be directly evaluated using the present MCD dataset. Older adults with atrial fibrillation have been reported to account for an increasing proportion of ischemic stroke mortality, especially in nonmetropolitan areas, underlining the need for equitable access to anticoagulation and stroke care (27, 31). Such gaps underscore continued needs to expand stroke care and preventive resources equitably. The continued decline in stroke mortality likely reflects the cumulative impact of improved hypertension and lipid management, reduced smoking prevalence, wider use of anticoagulation for atrial fibrillation, establishment of comprehensive stroke centers, increased availability of thrombolysis and mechanical thrombectomy, and advances in post-stroke rehabilitation. Collectively, these developments have improved stroke survival, thereby increasing the number of individuals living long enough to experience subsequent cognitive decline or die with coexisting neurodegenerative disease. Nevertheless, our study cannot determine the relative contribution of these individual factors because mortality records do not contain detailed clinical information.

Dementia, particularly AD, VaD, and unspecified dementia, has emerged as an increasing cause of mortality among older Americans. According to U. S. mortality data for 2018–2022, over 288,000 deaths in adults aged 65 above were primarily attributed to dementia (34). Possible contributors include better recognition, increased survival, and the interplay of vascular pathology with neurodegenerative changes reported in previous studies, although these mechanisms were not directly assessed in our analysis (39, 40). In addition, the observed increase likely reflects several concurrent phenomena, including continued population aging, improved survival from cardiovascular and cerebrovascular diseases, greater clinical awareness of dementia, expanded diagnostic evaluation, and evolving certification practices for recording dementia on death certificates. Therefore, increasing mortality should not necessarily be interpreted as indicating a proportional increase in disease incidence alone. In fact, an analysis from 2000 to 2020 reported that deaths where both AF and dementia were listed rose significantly, supporting shared mechanisms and a call for integrated approaches to management (18, 41). The overlap between stroke and dementia is biologically plausible because both conditions share several vascular risk factors; hypertension, diabetes, hyperlipidemia, atrial fibrillation, lifestyle factors, and genetic vulnerability such as APOE ε4 all contribute to vulnerability. At the population level, changes in vascular risk factor profiles may also have influenced these trends. Although improvements in smoking cessation and lipid management have reduced some cardiovascular risks, the increasing prevalence of obesity, diabetes mellitus, hypertension, and multimorbidity among older adults may continue to contribute to both cerebrovascular disease and cognitive decline.

Throughout the study period, “unspecified dementia” remained the leading contributor of stroke-associated dementia mortality, while VaD demonstrated a clear increase. One possible explanation is improved recognition and classification of vascular cognitive impairment; however, changes in death certificate coding practices, diagnostic awareness, and reporting over time may also have contributed to the observed patterns. Therefore, these findings should not be interpreted as evidence of true changes in disease occurrence alone. The predominance of unspecified dementia should also be interpreted cautiously, as it may reflect incomplete diagnostic characterization in older adults, limited access to specialist cognitive assessment, variability in physician certification practices, and changes in ICD-10 coding over time rather than true epidemiological differences between dementia subtypes. As diagnostic approaches and awareness have evolved, deaths that may previously have been recorded as unspecified dementia could increasingly be classified as AD or VaD. Consequently, temporal changes in subtype-specific mortality may partially reflect improvements in disease recognition and attribution rather than solely changes in the underlying burden of disease.

During the COVID-19 pandemic (2020–2021), mortality among persons with concurrent stroke and dementia increased before remaining higher during the post-COVID period (2022–2024), which may reflect excess mortality, disruption of routine healthcare services, delayed access to care, and the disproportionate impact of the pandemic on vulnerable populations (13, 42–44). Apart from demographic and healthcare system components, biological mechanisms have been proposed for the disproportionate mortality burden experienced as SARS-CoV-2 has been reported to exhibit neurovascular tropism via endothelial injury, hyperinflammatory cytokine cascades, and pro-thrombotic activation-all of which could potentially contribute to cerebrovascular injury and vascular cognitive impairment. However, our study cannot determine whether these mechanisms directly explain the observed mortality patterns (42). Furthermore, previous studies suggest that survivors with pre-existing dementia or cerebrovascular disease may remain more cognitively vulnerable due to accelerated neurodegenerative processes linked to post-infection microglial activation and chronic immune dysregulation. Pandemic-related disruptions may also have exacerbated the burden of multimorbidity by additional physiological stressors from pandemic-era disruptions in medication adherence, glycemic control, blood pressure management, lipid regulation, and stroke rehabilitation, which increased vulnerability to both recurrent stroke and dementia-related mortality (43). Older adults, women, and historically underserved populations appeared disproportionately affected, although our observational analysis cannot determine the relative contribution of biological vulnerability versus structural healthcare inequities. These factors should therefore be interpreted as plausible explanations supported by previous literature rather than direct conclusions from the present study (44). Additionally, temporary disruptions in healthcare delivery during the pandemic may have influenced death certification practices and diagnostic ascertainment, potentially affecting the reporting of both stroke and dementia on death certificates. This possibility should be considered when interpreting the observed increase during the pandemic period.

### 4.1 Limitations

Our interpretation has a number of significant limitations. First, mortality data can be misclassified, particularly when it comes to dementia. In addition, dementia-related deaths may be underestimated because dementia was identified using selected ICD-10 codes available within CDC WONDER, and some deaths may have been recorded under related dementia or neurodegenerative categories not included in the primary definition. The use of MCD data does not establish temporal or causal relationships between stroke and dementia and remains dependent on the completeness and accuracy of death certificate documentation (33). Furthermore, changes in diagnostic practices, physician awareness, ICD-10 coding, and death certification over the 25-year study period may have influenced the attribution of AD, VaD, and unspecified dementia on death certificates, thereby contributing to observed temporal trends independent of true changes in disease occurrence. Second, the use of national aggregated mortality data may obscure important regional and local heterogeneity. Third, substate or county-level heterogeneity may be obscured by our primary reliance on aggregated, national-level data. Fourth, comparability is limited because not all published studies of mortality control for the same covariates, such as comorbid illness, are comparable. Lastly, causal inference is complicated by the fact that many epidemiologic data sets only indirectly capture social determinants, such as wealth, education, and access to care. Finally, because this study relied on MCD records, the findings describe mortality patterns in which stroke and dementia were listed on death certificates and should not be interpreted as establishing causal relationships between these conditions. In addition, CDC WONDER does not include detailed clinical information regarding stroke severity, dementia stage, treatment received, imaging findings, medication use, or individual vascular risk factor control, precluding assessment of the mechanisms underlying the observed mortality trends.

## 5 Conclusion

Our findings demonstrate an evolving burden of deaths in which stroke and dementia were recorded among older Americans, highlighting important demographic and geographic disparities while recognizing the descriptive nature of death certificate data. The rising number of dementia-related deaths requires immediate attention as we commemorate decades of progress in lowering the death rates from strokes. Health gains are not distributed fairly, as evidenced by persistent disparities by sex, race, geography, and urbanization. Future public health initiatives must combine dementia prevention with vascular risk factor management while taking into consideration and resolving systemic disparities in care access. Investments in equitable acute and long-term care, community-level prevention, and policies targeted at lowering social and healthcare disparities will be necessary to achieve this. If current demographic and mortality patterns continue, the combined challenge of stroke and dementia demands a coordinated, multifactorial solution to reduce preventable deaths and protect quality of life. Beyond confirming previously reported mortality trends, our findings emphasize the growing coexistence of stroke with AD, VaD, and unspecified dementia in an aging U. S. population and highlight how demographic change, improvements in stroke survival, and persistent healthcare inequities are likely to shape the future burden of these conditions. These observations support the need for integrated cerebrovascular and cognitive health strategies that extend beyond acute stroke management to include long-term dementia prevention, equitable access to care, and healthy aging initiatives.

## Data Availability

The datasets presented in this study can be found in online repositories. The names of the repository/repositories and accession number(s) can be found at: https://wonder.cdc.gov/.

## References

[1] Murphy SJ Werring DJ. Stroke: causes and clinical features. Medicine (Baltimore). (2020) 48:561–6. doi: 10.1016/j.mpmed.2020.06.002, 32837228 PMC7409792

[2] Gale SA Acar D Daffner KR. Dementia. Am J Med. (2018) 131:1161–9. doi: 10.1016/j.amjmed.2018.01.022, 29425707

[3] Hassan W Javeid J Javeid A Bujala N Saim M Zahoor MM . Temporal trends of stroke and dementia-related mortality among older adults in the United States and impact of COVID-19: insight from 24-year analysis (S8.003). Neurology. (2025) 104:350. doi: 10.1212/WNL.0000000000208513

[4] Wu H Le Couteur DG Hilmer SN. Mortality trends of stroke and dementia: changing landscapes and new challenges. J American Geriatrics Society. (2021) 69:2888–98. doi: 10.1111/jgs.17322, 34133024

[5] Naveed MA Neppala S Chigurupati HD Rehan MO Ali A Naveed H . Trends in stroke-related mortality in atrial fibrillation patients in the United States: insights from the CDC WONDER database. Am Heart J. (2024) 49:100491. doi: 10.1016/j.ahjo.2024.100491, 39760108 PMC11696626

[6] Hemida MF Eldeeb A Saghir M Ibrahim AA Jairamani S Elbanna AF . Patterns and trends of mortality in patients with arrhythmia and cerebrovascular disease in the United States: a nationwide analysis of older adults. Sci Rep. (2026) 16:23611. doi: 10.1038/s41598-026-64412-z, 42533025 PMC13421678

[7] Feigin VL Brainin M Norrving B Martins SO Pandian J Lindsay P . World stroke organization: global stroke fact sheet 2025. Int J Stroke. (2025) 20:132–44. doi: 10.1177/17474930241308142, 39635884 PMC11786524

[8] Lackland DT Roccella EJ Deutsch AF Fornage M George MG Howard G . Factors influencing the decline in stroke mortality: a statement from the American Heart Association/American Stroke Association. Stroke. (2014) 45:315–53. doi: 10.1161/01.str.0000437068.30550.cf, 24309587 PMC5995123

[9] Pendlebury ST Rothwell PM. Prevalence, incidence, and factors associated with pre-stroke and post-stroke dementia: a systematic review and meta-analysis. Lancet Neurol. (2009) 8:1006–18. doi: 10.1016/S1474-4422(09)70236-4, 19782001

[10] Cao X Wang M Zhou M Mi Y Fazekas-Pongor V Major D . Trends in prevalence, mortality, and risk factors of dementia among the oldest-old adults in the United States: the role of the obesity epidemic. GeroScience. (2024) 46:4761–78. doi: 10.1007/s11357-024-01180-6, 38696055 PMC11336039

[11] Qiao Y Fayyaz AI Ding Y Ji X Zhao W. Recent advances in the prevention of secondary ischemic stroke: a narrative review. Brain Circulation. (2024) 10:283–95. doi: 10.4103/bc.bc_159_24, 40012589 PMC11850935

[12] Martin SS Aday AW Almarzooq ZI Anderson CAM Arora P Avery CL . Heart disease and stroke statistics—2024 update: a report from the American Heart Association. Circulation. (2024) 149. doi: 10.1161/CIR.0000000000001209 PMC12135016 36695182

[13] Axenhus M Frederiksen KS Zhou RZ Waldemar G Winblad B. The impact of the COVID-19 pandemic on mortality in people with dementia without COVID-19: a systematic review and meta-analysis. BMC Geriatr. (2022) 22:878. doi: 10.1186/s12877-022-03602-6, 36402953 PMC9675075

[14] Betts C Ahlfinger Z Udeh MC Kirmani BF. Recent updates on COVID-19 associated strokes. J Exp Neurosci. (2024) 19:26331055241287730. doi: 10.1177/26331055241287730, 39391859 PMC11465292

[15] CDC WONDER. Available online at: https://wonder.cdc.gov/ (Accessed November 30, 2025)

[16] CDC WONDER MCD. Available online at: https://wonder.cdc.gov/wonder/help/mcd.html (Accessed July 28, 2026)

[17] Saghir M Affan M Ahmed AM Mateen H Shoaib N Nabi R . Rising mortality and post-COVID shifts due to stroke in adults with dyslipidemia in the U.S. from 1999 to 2023: analysis of the CDC WONDER database. Int J Neurosci. (2026) 136:1–15. doi: 10.1080/00207454.2026.2639360, 41773527

[18] Saghir M Affan M Saghir E Qazi K Zafar M Rawal S . Rising dementia mortality among tobacco users in the US, 2005-2020: insights from CDC-WONDER database. Neurodegener Dis Manag. (2026):1–12. doi: 10.1080/17582024.2026.2647102, 41978491

[19] von Elm E Altman DG Egger M Pocock SJ Gøtzsche PC Vandenbroucke JP. The strengthening the reporting of observational studies in epidemiology (STROBE) statement: guidelines for reporting observational studies. J Clin Epidemiol. (2008) 61:344–9. doi: 10.1016/j.jclinepi.2007.11.008, 18313558

[20] Hemida MF Ibrahim AA Goel A Hussein M Patel K Sarfraz MR . Twenty-five years of angina–related mortality in elderly adults aged≥ 65 years: a retrospective cohort study using real-world data from the USA. ASIDE Internal Med. (2026) 2:33–42. doi: 10.71079/aside.im.110925248

[21] Aggarwal R Chiu N Loccoh EC Kazi DS Yeh RW Wadhera RK. Rural-urban disparities: diabetes, hypertension, heart disease, and stroke mortality among black and white adults, 1999-2018. J Am Coll Cardiol. (2021) 77:1480–1. doi: 10.1016/j.jacc.2021.01.032, 33736831 PMC8210746

[22] Ingram DD Franco SJ. 2013 NCHS Urban–Rural Classification Scheme for Counties.National Center for Health Statistics. 24776070

[23] Anderson RN Rosenberg HM. Age standardization of death rates: implementation of the year 2000 standard. Natl Vital Stat Rep. (1998) 47:1–16, 20. 9796247

[24] Joinpoint Regression Program. Available online at: https://surveillance.cancer.gov/joinpoint/ (Accessed November 30, 2025)

[25] Curtin C. S. Products - Data Briefs - Number 505. (2024)

[26] Faizan Ali M Ahmad H Fawzi Hemida M Faizan Tahir M Qadeer T Rasheed S . Aging, Alzheimer’s disease, and stroke: a 25-year longitudinal analysis of U.S. mortality trends. J Alzheimer's Dis. (2025) 108:616–28. doi: 10.1177/13872877251380689, 40990869 PMC13187842

[27] Wen J Hu J Zou G. Trends and differences in cardiovascular disease and Alzheimer’s disease-related mortality among older adults in the United States, 1999 to 2023: a CDC WONDER database analysis. Am Heart J Plus. (2025) 59:100618. doi: 10.1016/j.ahjo.2025.100618 41035497 PMC12483660

[28] QuickStats: Death Rates from Stroke among Persons Aged ≥65 Years, by Sex and Age Group — National Vital Statistics System, United States. (2018). 10.15585/mmwr.mm6933a5 PMC7439977 32817599

[29] Waqas SA Aamir J Ali D Imran Z Salim H Hassan A . Stroke mortality in the United States from 1968 to 2023: a CDC WONDER analysis. Int J Stroke. (1968) 21:559–68. 10.1177/17474930251384043 40976877

[30] McCandless MG Powers AY Baker KE Strickland AE. Trends in demographic and geographic disparities in stroke mortality among older adults in the United States. World Neurosurg. (2024) 185:e620–30. doi: 10.1016/j.wneu.2024.02.094 38403013

[31] Khan SA Assad AA Ashraf H Farooqi HA Abbasi SUAM Saleem H . National Trends in mortality due to ischemic stroke among older adults with atrial fibrillation in the USA, 1999-2020. Clin Cardiol. (2025) 48:e70115. doi: 10.1002/clc.70115, 40088054 PMC11909504

[32] QuickStats: Age-Adjusted Death Rates for Stroke among Adults Aged ≥ 65 Years, by Region and Metropolitan Status — National Vital Statistics System, United States, 2020 | MMWR. Available online at: https://www.cdc.gov/mmwr/volumes/71/wr/mm7143a4.htm (Accessed November 30, 2025)

[33] Steenland K MacNeil J Vega I Levey A. Recent trends in Alzheimer disease mortality in the United States, 1999 to 2004. Alzheimer Dis Assoc Disord. (2009) 23:165–70. doi: 10.1097/wad.0b013e3181902c3e, 19484918 PMC2719973

[34] Dementia Mortality in Adults Age 65 and Older: United States, 2018–2022. NCHS Data Brief. 10.15620/cdc/252450 PMC13266500 42269286

[35] Meyer JS Rauch GM Crawford K. Risk factors accelerating cerebral degenerative changes, cognitive decline and dementia. Int J Geriatr Psychiatry. (1999) 14:1050–61. 10607973 10.1002/(sici)1099-1166(199912)14:12<1050::aid-gps56>3.0.co;2-z

[36] Gottesman RF Schneider ALC Albert M Alonso A Bandeen-Roche K Coker L . Midlife hypertension and 20-year cognitive change: the atherosclerosis risk in communities neurocognitive study. JAMA Neurol. (2014) 71:1218–27. doi: 10.1001/jamaneurol.2014.1646, 25090106 PMC4226067

[37] Corrada MM Brookmeyer R Paganini-Hill A Berlau D Kawas CH. Dementia incidence continues to increase with age in the oldest old: the 90+ study. Ann Neurol. (2010) 67:114–21. doi: 10.1002/ana.21915, 20186856 PMC3385995

[38] Kleindorfer DO Towfighi A Chaturvedi S Cockroft KM Gutierrez J Lombardi-Hill D . 2021 guideline for the prevention of stroke in patients with stroke and transient ischemic attack: a guideline from the American Heart Association/American Stroke Association. Stroke. (2021) 52:e364–467. doi: 10.1161/STR.0000000000000375, 34024117

[39] Weiss J Puterman E Prather AA Ware EB Rehkopf DH. A data-driven prospective study of dementia among older adults in the United States. PLoS One. (2020) 15:e0239994. doi: 10.1371/journal.pone.0239994 33027275 PMC7540891

[40] Spence JD. Nutrition and risk of stroke. Nutrients. (2019) 11:647. doi: 10.3390/nu11030647, 30884883 PMC6470893

[41] Brookmeyer R Abdalla N Kawas CH Corrada MM. Forecasting the prevalence of preclinical and clinical Alzheimer’s disease in the United States. Alzheimers Dement. (2018) 14:121–9. doi: 10.1016/j.jalz.2017.10.009, 29233480 PMC5803316

[42] Shan D Xu Y Yang C Crawford TJ Holland C. COVID-19 infection associated with increased risk of new-onset vascular dementia in adults ≥50 years. Npj Dementia. (2025) 1:28. doi: 10.1038/s44400-025-00034-y

[43] Sim R Chong CW Loganadan NK Hussein Z Adam NL Lee SWH. Impact of COVID-19 lockdown on glycemic, weight, blood pressure control and medication adherence in patients with type 2 diabetes. PPA. (2023) 17:2109–17. doi: 10.2147/PPA.S420545, 37644964 PMC10461747

[44] Hua CL Cornell PY Zimmerman S Carder P Thomas KS. Excess mortality among assisted living residents with dementia during the COVID-19 pandemic. J Am Med Dir Assoc. (2022) 23:1743–1749.e6. doi: 10.1016/j.jamda.2022.07.023, 36065095 PMC9359515

