# Supplementary material for: Mortality patterns related to stroke and dementia among older adults in the U. S.: a CDC WONDER analysis from 1999 to 2024
Source: Front Neurol. 2026 Aug 26;17:1889772. doi: 10.3389/fneur.2026.1889772 (PMC13555609; doi:10.3389/fneur.2026.1889772)
Supplement: Supplementary file 1 [file Supplementary_file_1.docx]

**Supplementary File**

**Supplemental Figure 1:** Stroke and dementia**-**Related AAMRs per 100,000 Stratified by dementia sub-types in Older Adults in the United States, 1999-2024.

**Supplemental Table 1** Stroke and dementia–related Deaths, Stratified by Sex and Race, in Older Adults in the United States, 1999 to 2024.

| Year | Overall | Female | Male | NH American Indian or Alaska Native | NH Asian or Pacific Islander | NH Black or African American | NH White | Hispanic or Latino |
| --- | --- | --- | --- | --- | --- | --- | --- | --- |
| 1999 | 23,696 | 15,970 | 7,726 | 44 | 214 | 2,146 | 20,691 | 548 |
| 2000 | 36,314 | 24,249 | 12,065 | 65 | 441 | 3,353 | 31,537 | 840 |
| 2001 | 36,887 | 24,644 | 12,243 | 75 | 531 | 3,542 | 31,713 | 942 |
| 2002 | 38,885 | 25,904 | 12,981 | 99 | 560 | 3,782 | 33,315 | 1,041 |
| 2003 | 39,486 | 26,388 | 13,098 | 103 | 552 | 3,735 | 33,922 | 1,103 |
| 2004 | 38,466 | 25,612 | 12,854 | 109 | 564 | 3,830 | 32,703 | 1,211 |
| 2005 | 31,724 | 21,311 | 10,413 | 88 | 493 | 3,161 | 26,946 | 1,000 |
| 2006 | 28,238 | 18,726 | 9,512 | 77 | 546 | 2,802 | 23,753 | 1,027 |
| 2007 | 30,192 | 20,052 | 10,140 | 73 | 547 | 3,036 | 25,425 | 1,088 |
| 2008 | 30,269 | 20,135 | 10,134 | 70 | 562 | 2,979 | 25,382 | 1,230 |
| 2009 | 29,176 | 19,373 | 9,803 | 105 | 614 | 2,986 | 24,212 | 1,198 |
| 2010 | 30,720 | 20,381 | 10,339 | 103 | 667 | 3,149 | 25,450 | 1,306 |
| 2011 | 31,503 | 20,735 | 10,768 | 102 | 702 | 3,242 | 26,008 | 1,419 |
| 2012 | 32,288 | 21,146 | 11,142 | 110 | 765 | 3,290 | 26,525 | 1,538 |
| 2013 | 32,507 | 21,135 | 11,372 | 108 | 772 | 3,429 | 26,503 | 1,635 |
| 2014 | 33,377 | 21,730 | 11,647 | 154 | 816 | 3,397 | 27,068 | 1,878 |
| 2015 | 34,968 | 22,633 | 12,335 | 138 | 907 | 3,642 | 28,142 | 2,041 |
| 2016 | 36,414 | 23,512 | 12,902 | 148 | 963 | 3,712 | 29,350 | 2,181 |
| 2017 | 38,590 | 24,572 | 14,018 | 154 | 1,031 | 4,121 | 30,908 | 2,312 |
| 2018 | 39,747 | 24,990 | 14,757 | 174 | 1,109 | 4,133 | 31,703 | 2,568 |
| 2019 | 41,168 | 25,871 | 15,297 | 146 | 1,187 | 4,470 | 32,530 | 2,802 |
| 2020 | 50,387 | 31,499 | 18,888 | 181 | 1,554 | 5,867 | 39,019 | 3,707 |
| 2021 | 48,644 | 30,260 | 18,384 | 177 | 1,500 | 5,601 | 37,690 | 3,446 |
| 2022 | 50,906 | 31,661 | 19,245 | 179 | 1,536 | 5,666 | 39,520 | 3,744 |
| 2023 | 49,554 | 30,876 | 18,678 | 170 | 1,443 | 5,413 | 38,597 | 3,695 |
| 2024 | 51,687 | 31,866 | 19,821 | 201 | 1,564 | 5,789 | 39,983 | 3,885 |
| Total | 965,793 | 625,231 | 340,562 | 3,153 | 22,140 | 100,273 | 788,595 | 49,385 |

*Values represent the annual number of deaths related to stroke and dementia, stratified by Sex and Race, among older adults in the United States, extracted from the Centers for Disease Control and Prevention Wide-Ranging Online Data for Epidemiologic Research (CDC WONDER), 1999 to 2024; NH: Non-Hispanic.*

**Supplemental Table 2** Stroke and dementia–related Mortality, Stratified by Place of Death in Older Adults in the United States, 1999 to 2024.

| Place of Death | Deaths |
| --- | --- |
| Medical Facility - Inpatient | 142,921 |
| Medical Facility - Outpatient or ER | 17,175 |
| Medical Facility - Dead on Arrival | 1,686 |
| Medical Facility - Status unknown | 352 |
| Decedent's home | 195,620 |
| Hospice facility | 53,047 |
| Nursing home/long term care | 493,437 |
| Other | 59,777 |
| Place of death unknown | 1,778 |
| Total | 965,793 |

*Values represent the number of deaths related to stroke and dementia, stratified by place of death, among older adults in the United States, extracted from the Centers for Disease Control and Prevention Wide-Ranging Online Data for Epidemiologic Research (CDC WONDER), 1999 to 2024.*

**Supplemental Table 3** Annual percent change (APC) and average annual percent change (AAPC) of stroke and dementia–related Age-Adjusted Mortality Rates per 100,000 in Older Adults in the United States, 1999 to 2024.

|  |  |  | **Trend 1** |  | **Trend 2** |  | **Trend 3** |  | **Trend 4** |  | **Trend 5** |  |  |
| --- | --- | --- | --- | --- | --- | --- | --- | --- | --- | --- | --- | --- | --- |
|  | **Year** | **AAPC** | **Year** | **APC** | **Year** | **APC** | **Year** | **APC** | **Year** | **APC** | **Year** | **APC** |  |
| **Overall** | 1999-2024 | 1.09 (-0.77 to 2.99) | 1999-2001 | 24.72 (0.27 to 55.13) | 2001-2009 | −6.75 (-9.2 to -4.23) | 2009-2024 | 2.63 (1.82 to 3.44) |  |  |  |  |  |
| **Sex** |  |  |  |  |  |  |  |  |  |  |  |  |  |
| **Female** | 1999-2024 | 1.19 (-0.57 to 2.98) | 1999-2001 | 23.1 (0.02 to 51.51) | 2001-2010 | −5.6 (-7.56 to -3.59) | 2010-2024 | 2.89 (2.02 to 3.76) |  |  |  |  |  |
| **Male** | 1999-2024 | 1.06 (-0.9 to 3.08) | 1999-2001 | 25.18 (-0.8 to 57.98) | 2001-2009 | −7.24 (-9.8 to -4.61) | 2009-2024 | 2.83, 95% CI: 2.01 to 3.64 |  |  |  |  |  |
|  |  |  |  |  |  |  |  |  |  |  |  |  |  |
|  |  |  |  |  |  |  |  |  |  |  |  |  |  |
|  |  |  |  |  |  |  |  |  |  |  |  |  |  |
|  |  |  |  |  |  |  |  |  |  |  |  |  |  |
| **Race** |  |  |  |  |  |  |  |  |  |  |  |  |  |
| **NH American Indian** | 1999-2024 | 1.41 (-2.96 to 5.99) | 1999-2003 | 19.99 (5.04 to 37.07) | 2003-2006 | −12.1 (-37.88 to 24.37) | 2006-2024 | 0.05 (-0.92 to 1.04) |  |  |  |  |  |
| **NH White** | 1999-2024 | 1.11 (-0.67 to 2.94) | 1999-2001 | 24.11 (0.74 to 52.9) | 2001-2009 | −6.89 (-9.26 to -4.46) | 2009-2024 | 2.82 (2.01 to 3.63) |  |  |  |  |  |
| **NH Black or African American** | 1999-2024 | 1.01 (-1.59 to 3.68) | 1999-2002 | 18.12 (6.35 to 31.2) | 2002-2006 | −10.18 (-18.2 to -1.37) | 2006-2018 | −0.47 (-1.72 to 0.79) | 2018-2021 | 11.46 (-4.54 to 30.15) | 2021-2024 | −2.84 (-9.7 to 4.54) |  |
| **NH Asian or Pacific Islander** | 1999-2024 | 0.86 (-1.26 to 3.04) | 1999-2001 | 38.87 (12.39 to 71.59) | 2001-2006 | −8.35 (-12.74 to -3.75) | 2006-2018 | −0.95 (-1.82 to -0.08) | 2018-2021 | 8.76 (-1.27 to 19.81) | 2021-2024 | 4.16 (-8.82 to -0.21) |  |
| **Hispanic or Latino** | 1999-2024 | 2.13 (-0.27 to 4.59) | 1999-2003 | 10.6 (3.85 to 17.8) | 2003-2006 | −10.57 (-24.26 to 5.59) | 2006-2017 | 1.09 (0.005 to 2.2) | 2017-2020 | 10.88 (-0.25 to 23.25) | 2020-2024 | 0.72 (-2.11 to 3.64) |  |
| **Age Group** |  |  |  |  |  |  |  |  |  |  |  |  |  |
| **65-74 years** | 1999-2024 | 1.26 (-0.79 to 3.36) | 1999-2001 | 22.06 (-4.16 to 55.46) | 2001-2010 | −6.73 (-9.08 to -4.33) | 2010-2024 | 3.95 (2.98 to 4.92) |  |  |  |  |  |
| **75-84 years** | 1999-2024 | 0.62 (-1.29 to 2.57) | 1999-2001 | 22.95 (-1.53 to 53.43) | 2001-2009 | −6.52 (-9.12 to -3.84) | 2009-2024 | 1.89 (1.01 to 2.78) |  |  |  |  |  |
| **85+ years** | 1999-2024 | 1.37 (-0.38 to 3.16) | 1999-2001 | 24.14 (0.8 to 52.89) | 2001-2010 | −5.84 (-7.74 to -3.9) | 2010-2024 | 3.27 (2.45 to 4.09) |  |  |  |  |  |
| **Census Region** |  |  |  |  |  |  |  |  |  |  |  |  |  |
| **Northeast** | 1999-2024 | 0.14 (-1.48 to 1.81) | 1999-2001 | 23.46 (3.01 to 47.97) | 2001-2007 | −7.96 (-11.43 to -4.34) | 2007-2024 | 0.66 (0.06 to 1.27) |  |  |  |  |  |
| **Midwest** | 1999-2024 | 1.12 (-0.49 to 2.78) | 1999-2001 | 21.85 (0.46 to 47.74) | 2001-2011 | −5.04 (-6.58 to -3.47) | 2011-2024 | 3.14 (2.22 to 4.07) |  |  |  |  |  |
| **South** | 1999-2024 | 1.07 (-1.26 to 3.46) | 1999-2003 | 7.48 (1.8 to 13.47) | 2003-2006 | −13.24 (-26.5 to 2.39) | 2006-2017 | 0.18 (-1.1 to 1.49) | 2017-2021 | 9.52 (2.3 to 17.26) | 2021-2024 | 0.65 (−5.3 to -6.98) |  |
| **West** | 1999-2024 | 0.95 (-1.18 to 3.13) | 1999-2001 | 36.02 (5.52 to 75.32) | 2001-2009 | −8.17 (-10.81 to -5.46) | 2009-2024 | 2.05 (1.15 to 2.95) |  |  |  |  |  |
|  |  |  |  |  |  |  |  |  |  |  |  |  |  |
|  |  |  |  |  |  |  |  |  |  |  |  |  |  |
|  |  |  |  |  |  |  |  |  |  |  |  |  |  |
|  |  |  |  |  |  |  |  |  |  |  |  |  |  |
|  |  |  |  |  |  |  |  |  |  |  |  |  |  |
| **Urbanization** |  |  |  |  |  |  |  |  |  |  |  |  |  |
| **Urban areas** | 1999-2020 | 0.66 (-1.66 to 3.05) | 1999-2001 | 25.1 (-0.91 to 57.95) | 2001-2009 | −6.9 (-9.5 to -4.23) | 2009-2020 | 2.43 (1.05 to 3.83) |  |  |  |  |  |
| **Rural areas** | 1999-2020 | 0.68 (-1.24 to 2.65) | 1999-2002 | 13.15 (5.02 to 21.91) | 2002-2006 | −8.31 (-14.36 to -1.82) | 2006-2018 | −0.7 (-1.67 to 0.27) | 2018-2020 | 10.73 (-2.94 to 26.35) |  |  |  |

*Values represent the* annual percent change and average annual percent change of stroke and dementia–related age-adjusted mortality rates per 100,000*, among older adults in the United States, computed using join point regression software, 1999 to 2024; NH: Non-Hispanic.*

**Supplemental Table 4** Overall and Sex‐Stratified Stroke and dementia–related Age-Adjusted Mortality Rates per 100,000 in Older Adults in the United States, 1999 to 2024.

| **Year** | **Overall** | **Female** | **Male** |
| --- | --- | --- | --- |
| **1999** | 69.34 (68.45-70.22) | 70.28 (69.19-71.37) | 65.47 (63.98-66.95) |
| **2000** | 104.65 (103.57-105.73) | 105.21 (103.88-106.54) | 100.49 (98.67-102.31) |
| **2001** | 104.57 (103.51-105.64) | 105.59 (104.27-106.91) | 99.85 (98.06-101.65) |
| **2002** | 108.91 (107.83-109.99) | 109.99 (108.65-111.34) | 104.49 (102.67-106.32) |
| **2003** | 108.76 (107.68-109.83) | 110.61 (109.27-111.95) | 102.46 (100.68-1-4.24) |
| **2004** | 104.52 (103.48-105.57) | 106.56 (105.25-107.87) | 98.81 (97.09-100.54) |
| **2005** | 84.34 (83.42-85.27) | 86.85 (85.68-88.02) | 77.74 (76.23-79.24) |
| **2006** | 73.36 (72.5-74.21) | 75.05 (73.96-76.13) | 69.04 (67.65-70.44) |
| **2007** | 76.7 (75.84-77.57) | 78.77 (77.67-79.87) | 71.41 (70.01-72.8) |
| **2008** | 75.28 (74.43-76.13) | 77.78 (76.7-78.87) | 69.46 (68.1-70.82) |
| **2009** | 71.07 (70.25-71.88) | 73.63 (72.58-74.68) | 65.36 (64.07-66.66) |
| **2010** | 73.6 (72.77-74.42) | 76.3 (75.24-77.36) | 67.56 (66.25-68.86) |
| **2011** | 73.09 (72.28-73.9) | 75.68 (74.64-76.73) | 67.63 (66.35-68.91) |
| **2012** | 73.13 (72.33-73.93) | 75.52 (74.49-76.56) | 67.9 (66.63-69.16) |
| **2013** | 71.92 (71.13-72.7) | 74.22 (73.2-75.24) | 66.89 (65.66-68.12) |
| **2014** | 72.16 (71.38-72.94) | 74.81 (73.79-75.82) | 66.47 (65.26-67.68) |
| **2015** | 73.92 (73.14-74.7) | 76.5 (75.48-77.51) | 68.46 (67.24-69.67) |
| **2016** | 75.41 (74.63-76.19) | 78.07 (77.06-79.08) | 69.68 (68.47-70.88) |
| **2017** | 78.32 (77.53-79.11) | 80.59 (79.57-81.62) | 73.37 (72.15-74.59) |
| **2018** | 78.82 (78.04-79.59) | 80.56 (79.55-81.57) | 74.95 (73.74-76.17) |
| **2019** | 80.09 (79.32-80.87) | 82.36 (81.35-83.38) | 75.49 (74.29-76.7) |
| **2020** | 96.45 (95.61-97.3) | 99.05 (97.94-100.15) | 90.85 (89.54-92.15) |
| **2021** | 99.5 (98.61-100.39) | 102.63 (101.47-103.79) | 93.49 (92.12-94.85) |
| **2022** | 96.71 (95.87-97.55) | 98.54 (97.44-99.63) | 92.52 (91.2-93.84) |
| **2023** | 95.13 (94.29-95.97) | 99.1 (97.99-100.2) | 87.96 (86.68-89.24) |
| **2024** | 99.25 (98.39-100.11) | 102.4 (101.27-103.52) | 93.11 (91.8-94.42) |

*Values represent the annual* age-adjusted mortality rates-related to stroke and dementia per 100,000*, stratified by sex, among older adults in the United States, extracted from the Centers for Disease Control and Prevention Wide-Ranging Online Data for Epidemiologic Research (CDC WONDER), 1999 to 2024.*

**Supplemental Table 5** Stroke and dementia–related Age-Adjusted Mortality Rates per 100,000, Stratified by Race in older Adults in the United States, 1999 to 2024.

| **Year** | **NH American Indian or Alaska Native** | **NH Asian or Pacific Islander** | **NH Black** | **NH White** | **Hispanic or Latinos** |
| --- | --- | --- | --- | --- | --- |
| **1999** | 42.58 (30.81-57.35) | 36.54 (31.56-41.52) | 82.97 (79.45-86.49) | 69.88 (68.92-70.83) | 42.27 (38.69-45.85) |
| **2000** | 55.17 (42.39-70.59) | 70.04 (63.4-76.69) | 128.16 (123.82-132.51) | 105.12 (103.96-106.28) | 60.95 (56.79-65.12) |
| **2001** | 64.61 (50.73-81.11) | 76.9 (70.26-83.54) | 134.48 (130.04-138.92) | 104.23 (103.08-105.38) | 64.76 (60.58-68.95) |
| **2002** | 83.31 (67.48-101.74) | 76.44 (70.03-82.86) | 143.09 (138.52-147.67) | 108.37 (107.21-109.54) | 68.06 (63.87-72.25) |
| **2003** | 84.93 (68.28-101.59) | 69.57 (63.69-75.45) | 140.01 (135.51-144.52) | 108.75 (107.6-109.91) | 68.88 (64.77-73) |
| **2004** | 89.53 (72.52-106.53) | 67.22 (61.62-72.83) | 141.87 (137.37-146.38) | 103.76 (102.64-104.89) | 71.82 (67.72-75.92) |
| **2005** | 68 (54.32-83.09) | 54.24 (49.41-59.08) | 114.46 (110.46-118.47) | 83.94 (82.93-89.94) | 56.12 (52.61-59.64) |
| **2006** | 58.94 (46.36-73.88) | 56 (51.27-60.73) | 99.42 (95.73-103.12) | 72.49 (71.57-73.42) | 53.89 (50.56-57.22) |
| **2007** | 54.21 (42.33-68.37) | 52.51 (48.09-56.94) | 105.09 (101.34-108.84) | 76.18 (75.24-77.11) | 54.47 (51.21-27.73) |
| **2008** | 50.68 (39.36-64.25) | 51 (46.77-55.24) | 100.39 (96.78-104.01) | 74.77 (73.84-75.69) | 57.74 (54.5-60.99) |
| **2009** | 71.75 (57.87-85.63) | 52.11 (47.97-56.24) | 97.76 (94.25-101.28) | 70.18 (69.29-71.07) | 53.35 (50.32-56.39) |
| **2010** | 67.36 (54.14-80.58) | 54.49 (50.34-58.64) | 100.46 (96.94-103.98) | 72.77 (71.87-73.67) | 55.65 (52.62-58.68) |
| **2011** | 62.84 (50.49-75.19) | 51.65 (47.82-55.48) | 100.1 (96.65-103.56) | 72.51 (71.63-73.4) | 55.23 (52.35-58.11) |
| **2012** | 62.94 (51.02-74.86) | 52 (48.31-55.7) | 97.32 (93.98-100.66) | 72.68 (71.79-73.56) | 55.98 (53.17-58.78) |
| **2013** | 58.48 (47.32-69.64) | 48.35 (44.93-51.77) | 97.69 (94.4-100.97) | 71.46 (70.59-72.33) | 55.66 (52.95-58.37) |
| **2014** | 78.18 (65.67-90.68) | 47.38 (44.12-50.64) | 93.49 (90.32-96.65) | 71.9 (71.04-72.77) | 59.68 (56.97-62.39) |
| **2015** | 64.13 (53.25-75) | 48.65 (45.47-51.83) | 96.56 (93.4-99.71) | 73.7 (72.83-74.57) | 60.73 (58.08-63.37) |
| **2016** | 66.9 (56.01-77.8) | 48.55 (45.47-51.63) | 95.25 (92.16-98.34) | 75.73 (74.86-76.6) | 61.53 (58.93-64.12) |
| **2017** | 66.26 (55.71-76.82) | 48.27 (45.32-51.23) | 102.09 (98.94-105.23) | 78.75 (77.87-79.64) | 61.34 (58.83-63.86) |
| **2018** | 69.27 (58.88-79.66) | 49.02 (46.12-51.92) | 98.76 (95.72-101.8) | 79.42 (78.54-80.3) | 65.34 (62.8-67.89) |
| **2019** | 55.51 (46.42-64.59) | 49.65 (46.81-52.49) | 103.24 (100.19-106.3) | 80.44 (79.56-81.32) | 68.51 (65.96-71.06) |
| **2020** | 64.1 (54.67-73.54) | 61.21 (58.15-64.27) | 131.17 (127.78-134.56) | 95.68 (94.73-96.63) | 86.5 (83.7-89.3) |
| **2021** | 70.03 (59.59-80.46) | 61.56 (58.43-64.69) | 131.62 (128.13-135.12) | 100.76 (99.74-101.78) | 82.5 (79.73-85.27) |
| **2022** | 61.08 (52.03-70.13) | 56.92 (54.06-59.87) | 127.04 (123.69-130.39) | 97.87 (96.91-98.84) | 83.96 (81.25-86.66) |
| **2023** | 56.45 (47.91-64.99) | 52.17 (49.47-54.87) | 119.8 (116.57-123.04) | 97.49 (96.52-98.47) | 81.71 (79.06-84.36) |
| **2024** | 64.4 (55.4-73.4) | 56.29 (53.49-59.09) | 127.98 (124.64-131.33) | 101.08 (100.08-102.07) | 86.06 (83.34-88.78) |

*Values represent age-adjusted mortality rates per 100,000 for stroke and dementia, stratified by race, among older adults in the United States, extracted from the Centers for Disease Control and Prevention Wide-Ranging Online Data for Epidemiologic Research (CDC WONDER), 1999 to 2024.* NH, non-Hispanic.

**Supplemental Table 6** Stroke and dementia–related Crude Mortality Rates per 100,000, Stratified by Age Group in Older Adults in the United States, 1999 to 2024.

| **Year** | **Age 65-74 years** | **Age 75-84 years** | **Age 85+ years** |
| --- | --- | --- | --- |
| **1999** | 9.72 (9.27-10.17) | 69.5 (68.02-70.98) | 322.8 (317.33-328.26) |
| **2000** | 14.77 (14.21-15.32) | 104.83 (103.02-106.63) | 486.84 (480.2-493.48) |
| **2001** | 14.84 (14.29-15.4) | 103.07 (101.29-104.84) | 491.08 (484.47-497.7) |
| **2002** | 14.9 (14.34-15.45) | 105.72 (103.94-107.5) | 518.47 (511.72-525.22) |
| **2003** | 14.83 (14.27-15.38) | 107.05 (105.27-108.84) | 513.57 (506.92-520.22) |
| **2004** | 13.92 (13.39-14.46) | 103.88 (102.13-105.63) | 492.16 (485.71-498.61) |
| **2005** | 11.06 (10.58-11.53) | 82.01 (80.45-83.56) | 403 (397.26-408.74) |
| **2006** | 9.69 (9.25-10.13) | 72.62 (71.16-74.08) | 346.63 (341.4-351.87) |
| **2007** | 10.06 (9.61-10.5) | 75.69 (74.2-77.18) | 363.23 (357.97-368.49) |
| **2008** | 9.68 (9.25-10.1) | 74.64 (73.16-76.12) | 356.54 (351.4-361.67) |
| **2009** | 9.44 (9.02-9.85) | 70.71 (69.27-72.16) | 334.67 (329.78-339.57) |
| **2010** | 9.43 (9.02-9.84) | 73.26 (71.79-74.72) | 347.78 (342.85-352.71) |
| **2011** | 9.5 (9.1-9.9) | 73.04 (71.58-74.5) | 344.14 (339.34-348.94) |
| **2012** | 9.58 (9.19-9.97) | 71.83 (70.39-73.27) | 347.46 (342.7-352.22) |
| **2013** | 9.88 (9.49-10.27) | 70.84 (69.41-72.26) | 339.21 (334.57-343.86) |
| **2014** | 9.34 (8.97-9.71) | 69.69 (68.3-71.09) | 346.87 (342.22-351.52) |
| **2015** | 9.63 (9.27-10) | 70.41 (69.02-71.81) | 358.03 (353.35-362.71) |
| **2016** | 10.21 (9.84-10.58) | 70.02 (68.65-71.4) | 368.71 (364-373.42) |
| **2017** | 10.8 (10.43-11.17) | 73.07 (71.69-74.45) | 380.88 (376.13-385.64) |
| **2018** | 10.74 (10.38-11.11) | 73.31 (71.95-74.66) | 384.84 (380.09-389.59) |
| **2019** | 11.11 (1-.74-11.48) | 74.23 (72.9-75.57) | 390.86 (386.09-395.63) |
| **2020** | 13.86 (13.45-14.26) | 89.89 (88.45-91.34) | 466.9 (461.71-472.09) |
| **2021** | 13.84 (13.44-14.24) | 90.24 (88.78-91.71) | 491.32 (485.7-496.94) |
| **2022** | 14.33 (13.92-14.73) | 88.95 (87.56-90.35) | 469.94 (464.67-475.22) |
| **2023** | 13.77 (13.38-14.16) | 84.04 (82.71-85.36) | 473.64 (468.22-479.06) |
| **2024** | 14.16 (13.77-14.56) | 90.5 (89.12-91.88) | 486.72 (481.22-492.21) |

*Values represent crude mortality rates related* to stroke and dementia per 100,000*, stratified by age group, among older adults in the United States, extracted from the Centers for Disease Control and Prevention Wide-Ranging Online Data for Epidemiologic Research (CDC WONDER), 1999 to 2024.*

**Supplemental Table 7** Stroke and dementia–related Age-Adjusted Mortality Rates per 100,000, Stratified by Census Region in Older Adults in the United States, 1999 to 2024.

| **Year** | **Northeast** | **Midwest** | **South** | **West** |
| --- | --- | --- | --- | --- |
| **1999** | 51.83 (50.2-53.47) | 71.78 (69.97-73.59) | 79.74 (78.13-81.36) | 67.33 (65.34-69.31) |
| **2000** | 82.24 (80.2-84.29) | 108.18 (105.97-110.4) | 107.09 (105.23-108.95) | 120.87 (118.24-123.5) |
| **2001** | 77.22 (75.25-79.18) | 108.8 (106.59-111) | 108.11 (106.25-109.97) | 123.51 (120.89-126.13) |
| **2002** | 81.29 (79.29-83.29) | 112.58 (110.35-114.81) | 112.43 (110.54-114.31) | 128.56 (125.91-131.21) |
| **2003** | 77.51 (75.57-79.45) | 110.39 (108.2-112.58) | 113.92 (112.03-115.8) | 131.81 (129.16-134.45) |
| **2004** | 77.41 (75.48-79.34) | 106.15 (104.01-108.29) | 107.53 (105.72-109.35) | 126.53 (123.96-129.1) |
| **2005** | 61.06 (59.36-62.76) | 92.43 (90.45-94.41) | 90.05 (88.41-91.69) | 89.46 (87.33-91.58) |
| **2006** | 52.98 (51.41-54.55) | 79.06 (77.25-80.88) | 77.17 (75.67-78.67) | 81.35 (79.36-83.35) |
| **2007** | 56.8 (55.19-58.41) | 80.8 (78.98-82.63) | 82.25 (80.72-83.78) | 82.83 (80.84-84.81) |
| **2008** | 55.16 (53.58-56.74) | 80.39 (78.59-82.2) | 80.2 (78.71-81.7) | 81.43 (79.48-83.37) |
| **2009** | 52.52 (50.99-54.05) | 76.41 (74.66-78.16) | 76.06 (74.62-77.5) | 75.18 (73.34-77.03) |
| **2010** | 55.2 (53.64-56.77) | 76.69 (79.94-78.43) | 80.71 (79.24-82.18) | 76.22 (74.39-78.06) |
| **2011** | 55.67 (54.11-57.23) | 76.75 (75.02-78.48) | 79.18 (77.76-80.61) | 75.71 (73.91-77.5) |
| **2012** | 56.82 (55.25-58.38) | 75.62 (73.91-77.32) | 78.95 (77.55-80.36) | 76.22 (74.44-77.99) |
| **2013** | 54.93 (53.41-56.46) | 74.29 (72.62-75.97) | 77.91 (76.53-79.29) | 75.02 (73.28-76.75) |
| **2014** | 55.09 (53.57-56.62) | 76.51 (74.83-78.2) | 77.07 (75.72-78.42) | 75.1 (73.39-76.81) |
| **2015** | 56.57 (55.04-58.11) | 76.36 (74.68-78.03) | 80.07 (78.71-81.43) | 76.95 (75.24-78.65) |
| **2016** | 53.56 (52.07-55.04) | 76.76 (75.09-78.42) | 81.76 (80.41-83.11) | 83.04 (81.29-84.79) |
| **2017** | 54.92 (53.44-56.4) | 80.02 (78.33-81.71) | 84.77 (83.41-86.13) | 86.93 (85.15-88.7) |
| **2018** | 53.8 (52.35-55.26) | 80.07 (78.39-81.74) | 87.67 (86.31-89.04) | 85.2 (83.47-86.93) |
| **2019** | 55.75 (54.27-57.22) | 83.05 (81.36-84.74) | 88.97 (87.62-90.32) | 83.75 (82.06-85.45) |
| **2020** | 66.26 (64.66-67.86) | 101.76 (99.89-103.63) | 109.41 (107.93-110.89) | 95.55 (93.76-97.33) |
| **2021** | 63.1 (61.5-64.7) | 100.48 (98.56-102.4) | 117.29 (115.7-118.88) | 101.32 (99.42-103.23) |
| **2022** | 59.9 (58.39-61.41) | 97.32 (95.49-99.14) | 116.11 (114.59-117.62) | 95.52 (93.75-97.3) |
| **2023** | 58 (56.51-59.5) | 97.7 (95.86-99.55) | 114.81 (113.3-116.32) | 91.71 (89.96-93.45) |
| **2024** | 59.38 (57.86-60.89) | 106.65 (104.72-108.59) | 119.5 (117.96-121.05) | 92.52 (90.76-94.27) |

*Values represent the* age-adjusted mortality rates related to stroke and dementia per 100,000*, stratified by census region, among older adults in the United States, extracted from the Centers for Disease Control and Prevention Wide-Ranging Online Data for Epidemiologic Research (CDC WONDER), 1999 to 2024.*

**Supplemental Table 8** Stroke and dementia–related Age-Adjusted Mortality Rates per 100,000, Stratified by State in older adults in the United States, in 2024.

| **State** | **Age-adjusted mortality rate (95% CI)** |
| --- | --- |
| **Oregon** | 202.78 (197.38-208.19) |
| **Delaware** | 186.73 (176.45-197.01) |
| **Colorado** | 171.79 (167.03-176.55) |
| **Maryland** | 162.57 (158.48-166.66) |
| **Minnesota** | 151.25 (147.26-155.24) |
| **South Carolina** | 147.11 (142.91-151.31) |
| **Florida** | 133.88 (132.22-135.55) |
| **Tennessee** | 130.41 (126.87-133.95) |
| **Kentucky** | 129.37 (125.02-133.72) |
| **Washington** | 128.44 (125.06-131.82) |
| **Vermont** | 123.21 (113.04-133.38) |
| **North Carolina** | 120.42 (117.68-123.15) |
| **Alaska** | 114.23 (101.28-127.18) |
| **Virginia** | 113.04 (110.11-115.98) |
| **Wisconsin** | 113.04 (109.65-116.42) |
| **Michigan** | 112.78 (110.17-115.39) |
| **Idaho** | 110.35 (103.99-116.7) |
| **Ohio** | 109.45 (107.09-111.81) |
| **Oklahoma** | 106.73 (102.5-110.95) |
| **Nebraska** | 105.93 (100.13-111.73) |
| **Mississippi** | 103.3 (98.52-108.08) |
| **Texas** | 102.46 (100.74-104.18) |
| **Wyoming** | 102.28 (91.64-112.92) |
| **Indiana** | 101.77 (98.64-104.89) |
| **Arkansas** | 98.79 (94.32-103.25) |
| **Rhode Island** | 95.9 (88.99-102.81) |
| **District of Columbia** | 93.55 (83.17-103.92) |
| **West Virginia** | 88.63 (83.46-93.8) |
| **Maine** | 86.9 (81.29-92.51) |
| **Louisiana** | 85.42 (81.88-88.97) |
| **South Dakota** | 84.53 (77.05-92.01) |
| **Illinois** | 83.63 (81.61-85.65) |
| **Hawaii** | 83.14 (78.05-88.24) |
| **Georgia** | 82.54 (80.1-84.98) |
| **New Hampshire** | 81.03 (75.3-86.75) |
| **Utah** | 80.89 (75.99-85.79) |
| **Iowa** | 79.62 (75.9-83.35) |
| **Nevada** | 79.21 (74.29-83.5) |
| **California** | 79.11 (77.95-80.27) |
| **New Mexico** | 77.6 (72.93-82.28) |
| **Montana** | 75.63 (69.33-81.92) |
| **North Dakota** | 69.13 (61.92-76.34) |
| **Missouri** | 68.69 (66.11-71.27) |
| **Kansas** | 68.38 (64.59-72.17) |
| **Pennsylvania** | 66.75 (65.1-68.4) |
| **Alabama** | 63.97 (61.17-66.78) |
| **Connecticut** | 59.12 (56.13-62.11) |
| **Massachusetts** | 58.1 (55.91-60.3) |
| **New York** | 54.21 (52.97-55.44) |
| **Arizona** | 49.66 (47.7-51.62) |
| **New Jersey** | 47.38 (45.64-49.12) |

*Values represent the* age-adjusted mortality rates related to stroke and dementia per 100,000*, stratified by states, among older adults in the United States, extracted from the Centers for Disease Control and Prevention Wide-Ranging Online Data for Epidemiologic Research (CDC WONDER), 2024.*

**Supplemental Table 9** Stroke and dementia–related Age-Adjusted Mortality Rates per 100,000, Stratified by Urban-Rural Classification in Older Adults in the United States, 1999 to 2020.

| **Year** | **Urban Areas** | **Rural Areas** |
| --- | --- | --- |
| **1999** | 67.43 (66.45-68.4) | 77.08 (74.98-79.17) |
| **2000** | 103.73 (102.53-104.92) | 108.59 (106.11-111.06) |
| **2001** | 103.51 (102.33-104.7) | 108.92 (106.45-111.39) |
| **2002** | 107.52 (106.32-108.72) | 114.86 (112.33-117.4) |
| **2003** | 107.13 (105.94-108.31) | 115.53 (112.99-118.06) |
| **2004** | 102.92 (101.77-104.07) | 111.55 (109.06-114.03) |
| **2005** | 81.56 (80.55-82.57) | 96.46 (94.16-98.76) |
| **2006** | 71.21 (70.28-72.15) | 82.86 (80.74-84.97) |
| **2007** | 74.46 (73.51-75.4) | 86.72 (84.57-88.86) |
| **2008** | 72.47 (71.54-73.39) | 88.05 (85.89-90.21) |
| **2009** | 68.13 (67.25-69.02) | 84.66 (82.55-86.76) |
| **2010** | 70.9 (70-71.79) | 85.91 (83.8-88.01) |
| **2011** | 70.49 (69.61-71.37) | 85.34 (83.26-87.42) |
| **2012** | 70.92 (70.05-71.79) | 83.37 (81.33-85.42) |
| **2013** | 69.81 (68.96-70.67) | 82.05 (80.04-84.05) |
| **2014** | 69.83 (68.99-70.68) | 83.43 (81.42-85.45) |
| **2015** | 72.18 (71.33-73.03) | 82.28 (80.3-84.27) |
| **2016** | 74.26 (73.41-75.11) | 81.01 (79.05-82.97) |
| **2017** | 77.58 (76.72-78.44) | 81.83 (79.88-83.78) |
| **2018** | 78.13 (77.28-78.98) | 82.27 (80.34-84.21) |
| **2019** | 78.77 (77.92-79.61) | 86.6 (84.63-88.57) |
| **2020** | 95.68 (94.76-96.61) | 100.11 (98-102.22) |

*Values represent the* age-adjusted mortality rates-related to stroke and dementia per 100,000*, stratified by urban-rural classification, among older adults in the United States, extracted from the Centers for Disease Control and Prevention Wide-Ranging Online Data for Epidemiologic Research (CDC WONDER), 1999 to 2020.*

**Supplemental Table 10** Pandemic-related changes in age-adjusted mortality rates (AAMRs) and cause-specific stroke and dementia mortality rates by demographic group within the U.S, 1999-2024.

| **Variables** | **Pre-COVID AAMR (95% CI)** | **During-COVID AAMR (95% CI)** | **Post-COVID AAMR (95% CI)** | **% change(from Pre-COVID to During COVID)** | **% change(from During-COVID to Post-COVID)** | **% change(from Pre-COVID to Post-COVID)** |
| --- | --- | --- | --- | --- | --- | --- |
| **Overall** | 81.56 (81.37 to 81.74) | 97.88 (97.27 to 98.49) | 97.01 (96.53 to 97.5) | 20 | -0.88 | 18.94 |
| **Male** | 76.07 (75.77 to 76.37) | 92.06 (91.12 to 93) | 91.19 (90.43 to 91.94) | 21.02 | -0.94 | 11.8 |
| **Female** | 83.88 (83.64 to 84.12) | 100.76 (99.96 to 101.56) | 99.93 (99.3 to 100.57) | 20.12 | -0.82 | 19.13 |
| **Northeast** | 60.33 (59.98 to 60.69) | 64.7 (63.57 to 65.83) | 59.06 (58.19 to 59.93) | 7.24 | -8.71 | -2.1 |
| **Midwest** | 85.16 (84.76 to 85.56) | 101.08 (99.74 to 102.42) | 100.53 (99.45 to 101.61) | 18.69 | -0.54 | 18.04 |
| **South** | 87.28 (86.95 to 87.61) | 113.22 (112.14 to 114.3) | 116.82 (115.94 to 117.7) | 29.72 | 3.17 | 33.84 |
| **West** | 88.88 (88.44 to 89.31) | 98.32 (97.02 to 99.63) | 93.29 (92.28 to 94.31) | -2.76 | 7.94 | 4.96 |
| **Hispanic** | 60.14 (59.47 to 60.82) | 84.47 (82.5 to 86.43) | 83.88 (82.32 to 85.43) | 40.45 | -0.69 | 39.47 |
| **NH White** | 81.58 (81.37 to 81.79) | 98.27 (97.57 to 98.97) | 98.83 (98.27 to 99.4) | 20.45 | 0.56 | 21.14 |
| **NH Black or African American** | 106.69 (105.91 to 107.47) | 132.16 (129.72 to 134.61) | 124.95 (123.04 to 126.86) | 23.87 | -5.45 | 17.11 |
| **NH Asian or Pacific Islander** | 52.88 (52.02 to 53.74) | 60.59 (58.38 to 62.8) | 54.83 (53.21 to 56.45) | 14.58 | -9.5 | 3.68 |
| **NH American Indian or Alaska Native** | 65.33 (62.6 to 68.07) | 66.16 (59.01 to 73.31) | 60.7 (55.58 to 65.82) | 1.27 | -8.25 | -7.08 |
| **65-74 years** | 10.93 (10.83 to 11.02) | 13.85 (13.56 to 14.13) | 14.08 (13.86 to 14.31) | 26.71 | 1.66 | 28.81 |
| **75-84 years** | 80.05 (79.72 to 80.38) | 90.07 (89.04 to 91.1) | 87.81 (87.02 to 88.6) | 12.51 | -2.5 | 9.69 |
| **85+ years** | 386.87 (385.73 to 388.020 | 478.45 (474.63 to 482.26) | 476.66 (473.55 to 479.78) | 23.67 | -0.37 | 23.2 |

*Values represent the pandemic-related changes in age-adjusted mortality rates and cause-specific stroke and dementia mortality rates by demographic group, among older adults in the United States, 1999 to 2024.*
